# Soluble TREM2 ameliorates pathological phenotypes by modulating microglial functions in an Alzheimer’s disease model

**DOI:** 10.1038/s41467-019-09118-9

**Published:** 2019-03-25

**Authors:** Li Zhong, Ying Xu, Rengong Zhuo, Tingting Wang, Kai Wang, Ruizhi Huang, Daxin Wang, Yue Gao, Yifei Zhu, Xuan Sheng, Kai Chen, Na Wang, Lin Zhu, Dan Can, Yuka Marten, Mitsuru Shinohara, Chia-Chen Liu, Dan Du, Hao Sun, Lei Wen, Huaxi Xu, Guojun Bu, Xiao-Fen Chen

**Affiliations:** 1 Fujian Provincial Key Laboratory of Neurodegenerative Disease and Aging Research, Institute of Neuroscience, School of Medicine, Xiamen University, Xiamen, 361102 China; 2 Department of Traditional Chinese Medicine, School of Medicine, Xiamen University, Xiamen, 361102 China; 3 Xiamen Key Laboratory of Chiral Drugs, School of Medicine, Xiamen University, Xiamen, 361102 China; 4 Shenzhen Research Institute of Xiamen University, Shenzhen, 518063 China; 5 Department of Neuroscience, Mayo Clinic, Jacksonville, FL 32224 USA; 6 School of Medicine, Xiamen University, Xiamen, 361102 China; 7 Neuroscience Initiative, Sanford-Burnham-Prebys Medical Discovery Institute, La Jolla, CA 92037 USA

**Keywords:** Alzheimer's disease, Neuroimmunology

## Abstract

Triggering receptor expressed on myeloid cells 2 (TREM2) is a microglial surface receptor genetically linked to the risk for Alzheimer’s disease (AD). A proteolytic product, soluble TREM2 (sTREM2), is abundant in the cerebrospinal fluid and its levels positively correlate with neuronal injury markers. To gain insights into the pathological roles of sTREM2, we studied sTREM2 in the brain of 5xFAD mice, a model of AD, by direct stereotaxic injection of recombinant sTREM2 protein or by adeno-associated virus (AAV)-mediated expression. We found that sTREM2 reduces amyloid plaque load and rescues functional deficits of spatial memory and long-term potentiation. Importantly, sTREM2 enhances microglial proliferation, migration, clustering in the vicinity of amyloid plaques and the uptake and degradation of Aβ. Depletion of microglia abolishes the neuroprotective effects of sTREM2. Our study demonstrates a protective role of sTREM2 against amyloid pathology and related toxicity and suggests that increasing sTREM2 can be explored for AD therapy.

## Abstract (web summary)

TREM2 is a genetic risk factor for Alzheimer’s disease, and soluble TREM2 (sTREM2) in the CSF correlates with AD progression. Here the authors study the role of sTREM2 in a mouse model of Alzheimer’s disease, and find it reduces amyloid accumulation and increases the numbers of plaque-associated microglia which correlates with improved behavioural function in the mice.

## Introduction

Alzheimer’s disease (AD) is a progressive neurodegenerative disorder and the most prominent cause of dementia in the elderly population. The histopathological hallmarks of AD include the parenchymal deposition of amyloid-β (Aβ) plaques, the formation of tau neurofibrillary tangles, and neuroinflammation^1,2^. With the accumulation of these lesions in the brain, AD patients ultimately suffer from synaptic loss, neuronal death, and cognitive decline^3^. Although the pathogenic events for AD are likely multifactorial, genetic, pathological, and functional studies suggest that the disequilibrium between Aβ production and clearance is a contributing and perhaps a driving event leading to AD^4^. At present, enhancing the clearance of Aβ remains an attractive therapeutic or preventive strategy for AD^5^. Microglia are effective phagocytes in the central nervous system (CNS) for the uptake and proteolytic clearance of both soluble and fibrillary forms of Aβ^6^. Early studies have found that microglial cells are clustered around amyloid plaques in the brain of AD patients^7,8^ and AD mouse models^9^. Recently, plaque-associated microglia have been shown to constitute a protective barrier that compacts amyloid fibrils and reduces their toxicity^10,11^. The roles of microglia in AD have been further highlighted by recent genetic studies showing rare coding variants in several genes highly expressed in microglia as risk factors for late-onset AD^12–19^. Among them, a loss-of-function R47H mutation in the triggering receptor expressed on myeloid cells 2 (TREM2) constitutes one of the strongest single-allele genetic risk factors for AD^13,15,16^.

TREM2 is a type I transmembrane innate immune receptor predominantly expressed by microglia within the CNS^20,21^. The ectodomain of TREM2 has been reported to bind anionic and zwitterionic lipids^22^, apolipoproteins (including apoE, apoJ, and apoA) and lipoprotein particles^23–25^, and oligomeric Aβ as reported recently^26,27^. In the disease context, TREM2 plays important roles in microglial phagocytosis of apoptotic neurons, damaged myelin, and amyloid plaques^21,28^. TREM2 has also been shown to be essential for synaptic pruning in early development^29^. Furthermore, TREM2 regulates microglial biosynthetic metabolism^30^, proliferation^31^, survival^31^, cytokine release^32^, and their accumulation around plaques^11^. The impact of TREM2 on plaque accumulation was examined in *Trem2*-deficient AD mouse models, but the results were conflicting^22,33^. Interestingly, a later study suggested disease progression-dependent effects of TREM2 on amyloid pathology by demonstrating that *Trem2* deficiency ameliorates amyloid pathology early, but exacerbates it late in the disease process^34^.

TREM2 undergoes regulated proteolytic cleavage by ADAM10 and ADAM17 at H157–S158 peptide bond, resulting in the liberation of soluble TREM2 (sTREM2)^35–37^. sTREM2 is abundantly detected in human cerebrospinal fluid (CSF) and its levels are elevated in the CSF of patients with sporadic AD^38–41^. Interestingly, the levels of sTREM2 change dynamically during the progression of AD, peaking at the early symptomatic stages of the disease^38,40^. Importantly, the CSF concentrations of sTREM2 correlate with neuronal injury markers, including the CSF levels of total tau and phospho-tau; thus, they may serve as an immunomodulatory biomarker for neurodegeneration. Remarkably, sTREM2 was found to co-localize with neurons and plaques in vivo^42^, with functions remaining to be determined. We have recently reported that sTREM2 exerts functional roles in microglia by promoting inflammatory responses and shielding them from apoptosis^43^. However, whether sTREM2 also has protective effects against amyloid pathology and the related synaptic toxicity remains to be defined. In this study, we explored the effects of sTREM2 on pathological phenotypes in 5×FAD mouse model by direct stereotaxic injection of recombinant sTREM2 protein or by an adenovirus-associated virus (AAV)-mediated expression strategy. We found that sTREM2 reduces amyloid-β (Aβ) pathology and improves cognitive and synaptic functions by modulating microglial activity in 5×FAD mouse model, thus providing a rationale that sTREM2 can be explored for AD therapy.

## Results

### sTREM2 reduces plaque load and the associated toxicity

**Fig. 1:**
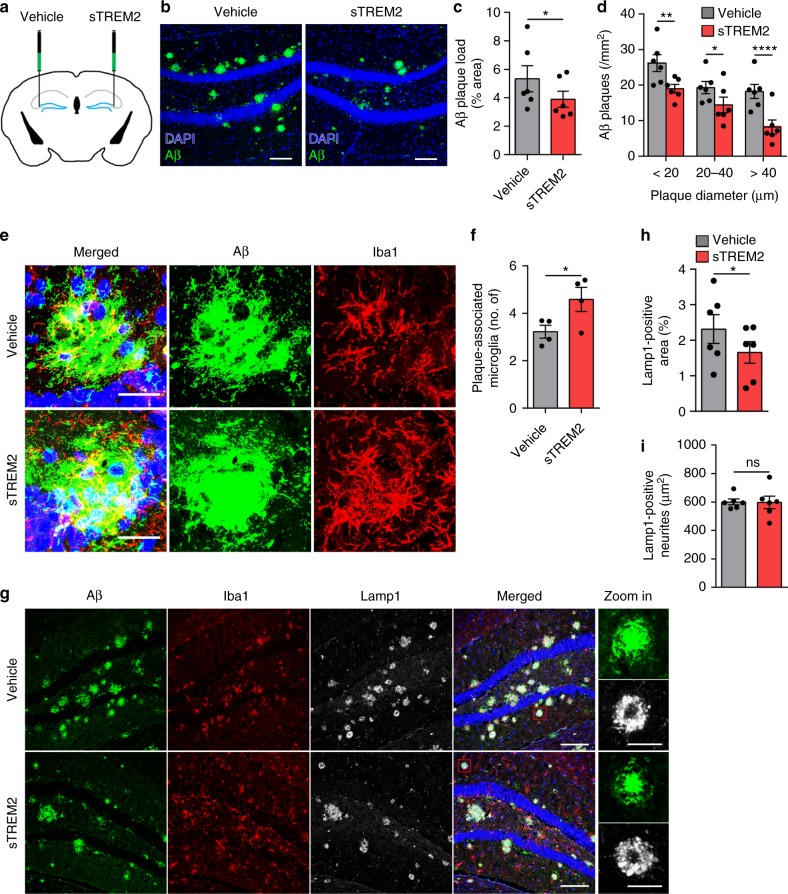
sTREM2 reduces amyloid plaque deposition and increases the number of plaque-associated microglia. **a** The 5×FAD mice at 7-month-old age were injected with vehicle (PBS) in the left hemisphere and 6 μg of sTREM2 protein in the right hemisphere. **b** Coronal sections of vehicle- or sTREM2-injected 5×FAD mice were stained with DAPI (blue) for nuclei and MOAB-2 (green) for Aβ. Representative z-stack images of the hippocampus regions are shown. Original magnification ×20; scale bar, 100 μm. **c** Quantitation of amyloid plaque deposition in **b** (*n* = 6 mice, 28 fields of each group for analysis, paired Student’s *t* test). **d** Quantitation of the number of plaques with different sizes in **b** (*n* = 6 mice, 28 fields of each group for analysis, paired Student’s *t* test). **e** Coronal sections from sTREM2-injected 5×FAD mice were stained with DAPI (blue) for nuclei, MOAB-2 (green) for Aβ, and Iba1 (red) for microglia. Representative z-stack images of the hippocampus regions are shown. Scale bar, 25 μm. **f** Quantitation of the number of plaque-associated microglia in **e** (*n* = 4 mice, 58 plaques of vehicle and 41 plaques of sTREM2 for analysis, paired Student’s *t* test). Plaques with 50 μm in diameter were selected for analysis. **g** Coronal sections from sTREM2-injected 5×FAD mice were stained with DAPI (blue) for nuclei, MOAB-2 (green) for Aβ, Iba1 (red) for microglia, and Lamp1 (white) for dystrophic neurites. Representative z-stack images of the hippocampus regions are shown. Original magnification ×20; scale bar, 100 μm. Zoom-in images on the right with a scale bar equal to 25 μm. **h** Quantitation of the total area of Lamp1-positive dystrophic neurites in **g** (*n* = 6 mice, 20 fields of each group for analysis, paired Student’s *t* test). **i** Quantitation of the area of Lamp1-positive dystrophic neurites within each plaque in **g** (*n* = 6 mice, 40 plaques of vehicle and 41 plaques of sTREM2 for analysis, paired Student’s *t* test). All data are presented as mean ± SEM. *p < 0.05; **p < 0.01; ****p < 0.0001; ns, not significant

We had previously developed a mammalian expression and purification system for recombinant sTREM2 protein (Supplementary Fig. 1a)^43^. To assess the impact of sTREM2 on AD-related pathology, we injected a recombinant sTREM2 protein into the right hippocampus of 5×FAD mice, with phosphate-buffered saline (PBS) injected into the left hippocampus as a vehicle control (Fig. 1a). Twenty-four hours after stereotaxic injection of sTREM2, immunofluorescence staining with human TREM2 antibody showed a sTREM2 distribution pattern that is diffused but limited to the right hippocampus (Supplementary Fig. 1b). However, the sTREM2 signal decayed thereafter and was barely detectable on day 3 and 7 after injection. Seven days after delivery to the hippocampus of 5×FAD mice, sTREM2 dramatically reduced the amyloid plaque load in the ipsilateral hippocampus (Fig. 1b, c). Of note, the number of large plaques (>40 μm in diameter) was markedly reduced by sTREM2 (*P* < 0.0001, paired Student’s *t* test) (Fig. 1d).

In addition to the amount of Aβ accumulation, the degree of plaque compaction plays a crucial role in determining the neurotoxicity of the plaque. It has been suggested that microglia clustered around the amyloid plaque constitute a barrier which impacts plaque composition and toxicity^10^. To gain insight into the effects of sTREM2 on microglial barrier function, we quantified the number of microglia in the vicinity of an amyloid plaque and found a significant increase upon sTREM2 administration (Fig. 1e, f). Consistent with its protective effects on plaque deposition, sTREM2 significantly reduced the total area of dystrophic neurites, as detected by immunostaining with an antibody against Lamp1 (Fig. 1g, h). However, no significant differences were observed between vehicle and sTREM2 treatment when calculating the amount of dystrophic neurites per plaque (Fig. 1i). Thus, distinct from the full-length TREM2, sTREM2 is unlikely involved in compacting amyloid fibrils to reduce their toxicity^11^. Collectively, these data reveal that sTREM2 protein reduces amyloid plaque deposition and enhances the clustering of microglia in the vicinity of the plaque, thereby reducing plaque load and the plaque-associated neurotoxicity.

### sTREM2 promotes microglial proliferation and migration

**Fig. 2:**
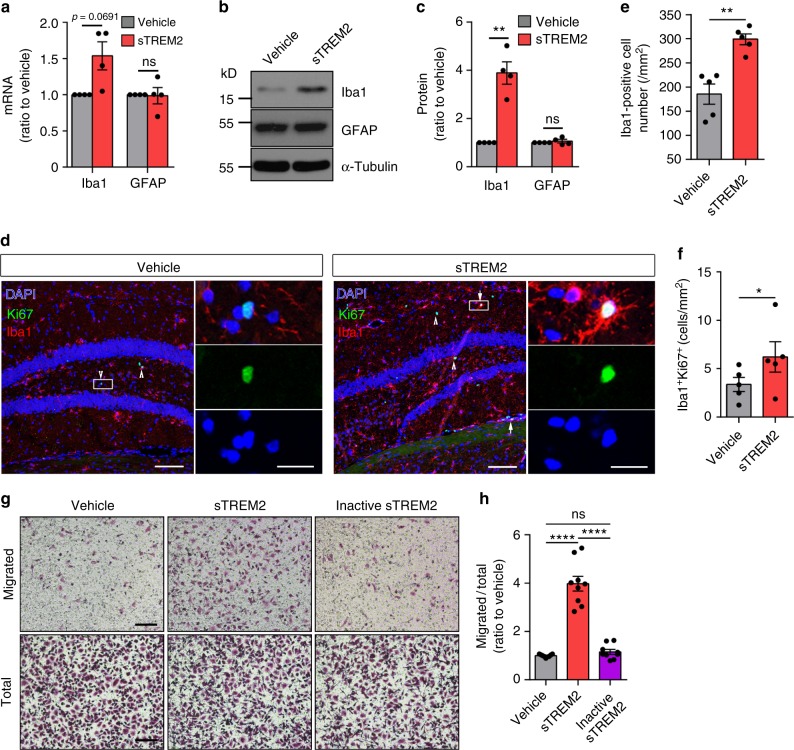
sTREM2 promotes microgliosis in 5×FAD mice and microglial migration. **a** The hippocampus was dissected from vehicle- or sTREM2-injected 5×FAD mice. After RNA extraction, the relative mRNA levels of Iba1 and GFAP in the hippocampus shown as a bar graph were determined by quantitative real-time PCR. β-actin was used as an internal control (*n* = 4 mice per group, paired Student’s *t* test). **b** Iba1 and GFAP proteins were analyzed by Western blotting 7 days after sTREM2 injection to the hippocampus of 5×FAD mice. **c** Quantitation of the protein levels of Iba1 and GFAP in **b** (*n* = 4 mice per group, paired Student’s *t* test). **d** Coronal sections from injected 5×FAD mice were stained with DAPI (blue) for nuclei, Ki67 (green) for proliferating cells, and Iba1 (red) for microglia. Representative z-stack images of the hippocampus regions are shown. Original magnification ×20; scale bar, 100 μm. Images on the right represent enlarged Ki67-positive cells with a scale bar equal to 20 μm. **e** Quantitation of the number of Iba1-positive cells in **d** (*n* = 5 mice, 22 fields of each group for analysis, paired Student’s *t* test). **f** Quantitation of the number of proliferating microglial cells, as indicated by the co-staining of Ki67 and Iba1 (Iba1^+^Ki67^+^) (*n* = 5 mice, 22 fields of each group for analysis, paired Student’s *t* test). **g** Primary microglial cells (10^5^) from WT mice were plated onto transwell chamber inserts. Following 24-h incubation with vehicle (PBS), native sTREM2 protein (100 nM), or heat-inactivated sTREM2 protein (100 nM), cells migrated through the membrane were stained with hematoxylin and eosin and imaged under a Nikon inverted microscope. Scale bar, 100 µm. **h** Quantitation of the number of migrated cells (*n* = 9 from three independent experiments, one-way ANOVA). All data are presented as mean ± SEM. *p < 0.05; **p < 0.01; ****p < 0.0001; ns, not significant

Proliferation and activation of microglia around the sites of amyloid plaque deposition is a prominent feature of AD^44^. The ionized calcium-binding adapter molecule 1 (Iba1) is widely employed as an immunohistochemical marker for both ramified and activated microglia, and its expression is increased upon microglial activation^45,46^. We found that the expression of Iba1 in the hippocampus of 5×FAD mice was significantly increased at the protein level, and a trend toward increased mRNA level upon sTREM2 administration (Fig. 2a–c). However, no significant differences were detected for the astrocytic marker GFAP. Staining of brain sections further revealed that sTREM2 administration led to a significantly higher number of proliferating microglia (Iba1^+^/Ki67^+^) and the total number of microglia (Iba1^+^) in the hippocampus (Fig. 2d–f). Interestingly, the increased Iba1 staining induced by sTREM2 on day 1 was sustained throughout day 7, even though sTREM2 was barely detectable on day 3 and 7 after injection (Supplementary Fig. 1b). In addition to Iba1, the expression levels of pro-inflammatory cytokines, including IL-1β and TNF, were significantly increased in the presence of sTREM2 (Supplementary Fig. 2a), consistent with our previous report that sTREM2 promotes microglial activation^43^. Importantly, both the pro-inflammatory cytokine IL-1β and the immune-activating molecule lipopolysaccharide enhanced the production of sTREM2 in primary microglia, as measured by enzyme-linked immunosorbent assay (ELISA); while the anti-inflammatory cytokine IL-10 had a minimal effect (Supplementary Fig. 2b). Therefore, sTREM2 and the pro-inflammatory cytokines appear to form a positive feedback loop, resulting in sustained microglial activation.

Since sTREM2 increases the number of plaque-associated microglia, we further tested a hypothesis that sTREM2 might act as a chemoattractant to promote microglial migration. Primary microglia isolated from wild-type (WT) mice were subjected to the transwell migration assay in the presence or absence of sTREM2. Interestingly, treatment with native but not heat-inactivated sTREM2 led to increased migration of microglia, as compared with the vehicle control (Fig. 2g, h). Our data hence suggest that sTREM2 not only enhances microgliosis in 5×FAD mice, but also acts as a chemoattractant for microglia.

### sTREM2 promotes microglial phagocytosis and clearance of Aβ

**Fig. 3:**
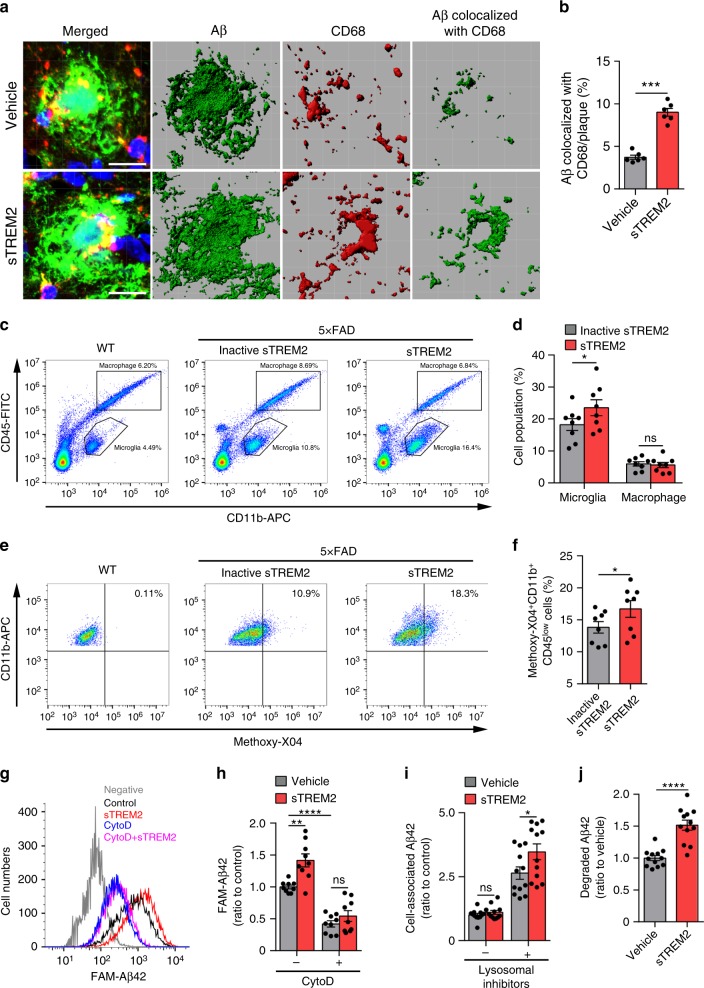
sTREM2 enhances microglial phagocytosis and degradation of Aβ. **a** Coronal sections from sTREM2-injected 5×FAD mice were stained with DAPI (blue) for nuclei, MOAB-2 (green) for Aβ, or CD68 (red) for a phagocytic phenotype of microglia. Three-dimensional reconstruction of Aβ, CD68, and Aβ colocalized with CD68 was shown. Scale bar, 25 μm. **b** Quantitation of the ratio of Aβ colocalized with CD68 within each plaque (*n* = 6 mice, 42 plaques of vehicle and 30 plaques of sTREM2 for analysis, paired Student’s *t* test). **c** Representative FACS dot plots of microglia or macrophage from WT or 5×FAD mice are shown. Microglia and microphage were identified as CD11b^+^CD45^low^ and CD11b^+^CD45^high^, respectively. **d** Quantitation of the cell population of microglia or macrophage in **c** (*n* = 8 mice, paired Student’s *t* test). **e** Representative FACS dot plots showing the microglial phagocytosis of Aβ in 5×FAD mice. **f** Quantification of the percentage (%) of methoxy-X04^+^CD11b^+^CD45^low^ hippocampal microglial cells by flow cytometry isolated from sTREM2-injected 5×FAD mice (*n* = 8 mice, paired Student’s *t* test). **g** Representative histogram displaying background fluorescence (negative) of untreated microglia and the distribution of FAM-Aβ42-positive cells treated with vehicle or sTREM2 protein. **h** Quantitation of the mean fluorescence in **g** to indicate the amount of FAM-Aβ42 uptake. Values were normalized to the vehicle control without cyto D (*n* = 9 from three independent experiments, two-way ANOVA, Bonferonni post hoc analyses). **i** The amounts of cell-associated Aβ42 were quantified by ELISA (*n* = 12 from four independent experiments, two-way ANOVA, Bonferonni post hoc analyses). **j** The amounts of degraded Aβ42 were calculated by subtracting the cell-associated Aβ in the absence of inhibitors from that in the presence of inhibitors (*n* = 12 from four independent experiments, unpaired Student’s *t* test). All data are presented as mean ± SEM. *p < 0.05; **p < 0.01; ***p < 0.001; ****p < 0.0001; ns, not significant

Microglia clustering in the vicinity of plaques is interpreted as an attempt to clear the pathological deposits of Aβ through phagocytosis and degradation. Indeed, quantitative assessment of the area of colocalization of Aβ with CD68, a phagocytic marker for microglia in the brain, revealed a significant increase in sTREM2-injected 5×FAD mice (Fig. 3a, b), suggesting that sTREM2 likely promotes phagocytic Aβ uptake by microglia. To further test this in vivo, we administered intraperitoneally methoxy-X04 3 h prior to microglial isolation and analyzed the proportion of methoxy-X04-positive microglial cells by flow cytometry. Comparing PBS-injected WT mice and 5×FAD mice treated with native or heat-inactivated sTREM2 revealed a significant increase in the number of CD11b^+^ CD45^low^ resident microglia in sTREM2-administered 5×FAD mice, while no significant change was observed for the number of CD11b^+^CD45^high^ peripheral macrophage (Fig. 3c, d). Therefore, the increased number of Iba1-positive cells upon sTREM2 treatment is likely contributed by resident microglia rather than infiltrating macrophages. A significant increase in the proportion of methoxy-X04-positive microglial cells was observed for 5×FAD mice injected with native sTREM2 (Fig. 3e, f), further demonstrating that sTREM2 enhances microglial phagocytosis of Aβ in vivo.

We further examined the effects of sTREM2 on Aβ uptake and/or degradation in cultured microglial cells. Primary postnatal microglia isolated from WT mice were incubated with fluorescently FAM-labeled Aβ42 (FAM-Aβ42). We found an increase in FAM-Aβ42 uptake upon sTREM2 stimulation (Fig. 3g, h). However, the effect was diminished in the presence of a phagocytosis inhibitor cytochalasin D. To further investigate the role of sTREM2 in microglial Aβ endocytic trafficking, we incubated microglial cells with Aβ, together with vehicle control or sTREM2 in the presence or absence of lysosomal enzyme inhibitors (Pepstatin A, Leupeptin, and E-64d) or chloroquine. Cell-associated Aβ levels were significantly increased by sTREM2 in the presence of lysosomal inhibitors (Fig. 3i and Supplementary Fig. 3a). When lysosomal degradation of Aβ was calculated by subtracting the cell-associated Aβ in the absence of inhibitors from that in the presence of inhibitors, we found that sTREM2 significantly enhanced Aβ lysosomal degradation, as compared with vehicle control (Fig. 3j and Supplementary Fig. 3b).

Since the steady-state levels of brain Aβ represent a dynamic equilibrium between its rate of clearance and production from the amyloid precursor protein (APP), we further explored the molecular mechanism by which sTREM2 reduces Aβ levels. We performed a series of experiments to evaluate whether sTREM2 induces changes in the levels of APP, its cleavage products, or Aβ-degrading enzymes in sTREM2-injected 5×FAD mice. No significant changes in the levels of full-length APP, α-CTF, or β-CTF were detected upon sTREM2 injection in 5×FAD mice (Supplementary Fig. 3c, d). Quantitative real-time polymerase chain reaction (PCR) of the transcript levels of Aβ-degrading enzymes, including insulin-degrading enzyme (IDE), neprilysin (NEP), endothelin-converting enzyme 1 (ECE1), matrix metalloproteinase 2 (MMP2), and matrix metalloproteinase 9 (MMP9), did not detect any significant alterations in response to sTREM2 treatment (Supplementary Fig. 3e). Taken together, we provide both in vivo and in vitro evidence that sTREM2 promotes microglial phagocytosis and clearance of Aβ, which may account for sTREM2-mediated reduction of Aβ deposition in the brain of 5×FAD mice.

### Microglia are essential for the protective effects of sTREM2

**Fig. 4:**
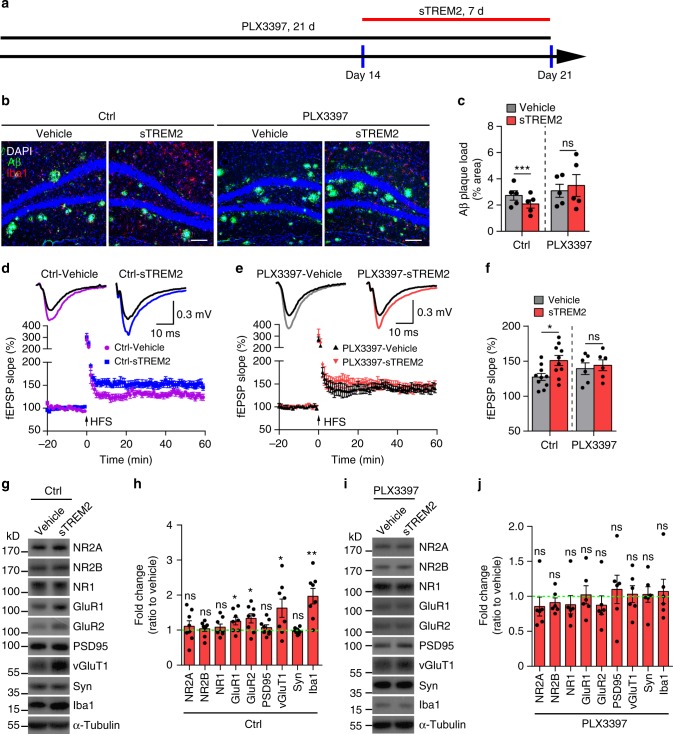
Depletion of microglia attenuates the protective effects of sTREM2 on Aβ pathology and synaptic plasticity. **a** Scheme for PLX3397 administration and sTREM2 injection in 5×FAD mice. **b** The 5×FAD mice were fed with either PLX3397 or control chow for 14 days and then injected with either sTREM2 protein or vehicle control. After continued feeding with PLX3397 or control chow for 7 days, coronal sections were stained with DAPI (blue) for nuclei, MOAB-2 (green) for Aβ, and Iba1 (red) for microglia. Representative images of the hippocampus region are shown. Original magnification ×20; scale bar, 100 μm. **c** Quantitation of amyloid plaque deposition in **b** (*n* = 5 mice, 18 fields of ctrl and 22 fields of PLX3397 for analysis, paired Student’s *t* test). **d**, **e** Brain slices from control chow (**d**) or PLX3397-fed (**e**) 5×FAD mice were incubated with 50 nM recombinant sTREM2 protein for 1 h at room temperature, and transferred to the chamber for LTP recording. Time course of fEPSP measures were recorded in the hippocampal CA1 region before and after 100-Hz stimulation in the Schaffer collateral region. Normalized fEPSP slopes were plotted every 1 min for each group. **f** The averaged fEPSPs recorded 50–60 min after induction of LTP (*n* = 10 slices for Ctrl, *n* = 6 slices for PLX3397, four mice per group, unpaired Student’s *t* test). **g** Synaptic proteins from the hippocampi were analyzed by Western blotting 7 days after vehicle or sTREM2 protein injection to the 5×FAD mice fed with control chow. **h** Quantitation of Western blots in **g** (*n* = 8 mice per group, paired Student’s *t* test). **i** Synaptic proteins from the hippocampi were analyzed by Western blotting 7 days after vehicle or sTREM2 protein injection to the 5×FAD mice fed with PLX3397. **j** Quantitation of Western blots in **i** (*n* = 6 mice per group, paired Student’s *t* test). All data are presented as mean ± SEM. *p < 0.05; **p < 0.01; ***p < 0.001; ns, not significant

Both our current and previous studies pinpoint a critical role of sTREM2 in modulating microglial function^43^. To determine whether microglia mediate the protective effects of sTREM2 on amyloid pathology, we employed the CSF1R inhibitor PLX3397 to deplete microglia from 5×FAD mice^47,48^. Following 14 days of PLX3397 treatment, microglial number as assessed by Iba1 staining was substantially reduced, with efficiency comparable with the previous work in 5×FAD mice (Supplementary Fig. 4a, b)^48^. Consistent with previous reports that microglial elimination in 5×FAD mice prevents neuronal loss^48^, increased levels of synaptic markers (including NR2A, NR1, GluR1, and vGluT1) were observed in PLX3397 administered to 5×FAD mice (Supplementary Fig. 4c, d). However, no significant changes in the levels of full-length APP, α-CTF, or β-CTF were detected upon PLX3397 treatment. To determine if microglia are responsible for sTREM2-mediated plaque reduction, we depleted microglia with 14 days of PLX3397 treatment, followed by administering sTREM2 to the hippocampus of 5×FAD mice (Fig. 4a). In contrast to the control without PLX3397 treatment, the plaque load was no longer reduced by sTREM2 in 5×FAD mice treated with PLX3397, suggesting an indispensable role of microglia in sTREM2-mediated plaque reduction (Fig. 4b, c).

Next, we studied the impact of sTREM2 administration on hippocampal synaptic plasticity by inducing long-term potentiation (LTP) in the Schaffer collateral (SC) pathway with high-frequency stimulation. Intriguingly, the LTP impairment of 5×FAD mice was ameliorated when acute hippocampal slices obtained from 5×FAD mice were pre-incubated with sTREM2 protein as compared with the vehicle control (Fig. 4d, f). More interestingly, such effects were diminished in microglia-depleted 5×FAD mice (Fig. 4e, f), suggesting that microglia mediate the protective functions of sTREM2 in synaptic plasticity. In contrast to the 5×FAD mice, sTREM2 had minimal effects on the LTP in either microglia-intact or microglia-eliminated WT mice (Supplementary Fig. 4e–g). To better understand synaptic alterations upon sTREM2 administration, we examined the levels of various proteins involved in synaptic plasticity and function. The levels of postsynaptic glutamate (AMPAR) receptor subunits (GluR1 and GluR2) and the presynaptic vesicular glutamate transporter 1 (vGluT1) were significantly increased upon sTREM2 treatment in the hippocampus (Fig. 4g, h). However, sTREM2 could no longer increase the levels of these proteins in the absence of microglia (Fig. 4i, j).

Recently, sTREM2 was found to co-localize with neurons in vivo^42^. To further investigate whether neurons directly respond to sTREM2, primary neuronal cultures at DIV 12–14 were incubated with sTREM2 and subjected to whole-cell patch-clamp experiments to record miniature excitatory postsynaptic currents (mEPSCs) and miniature inhibitory postsynaptic currents (mIPSCs). The amplitude and frequency of both mEPSCs and mIPSCs did not significantly differ between Fc- and sTREM-Fc-treated groups (Supplementary Fig. 4h–k). Furthermore, sTREM2 did not affect the abundance of synaptic proteins in the primary neuronal cultures (Supplementary Fig. 4l, m), suggesting that sTREM2 does not directly modulate synaptic plasticity and function. Taken together, these data indicate that microglia play an indispensable role in mediating the protective effects of sTREM2 on amyloid plaque pathology and synaptic plasticity.

### Validation with physiologically produced sTREM2

**Fig. 5:**
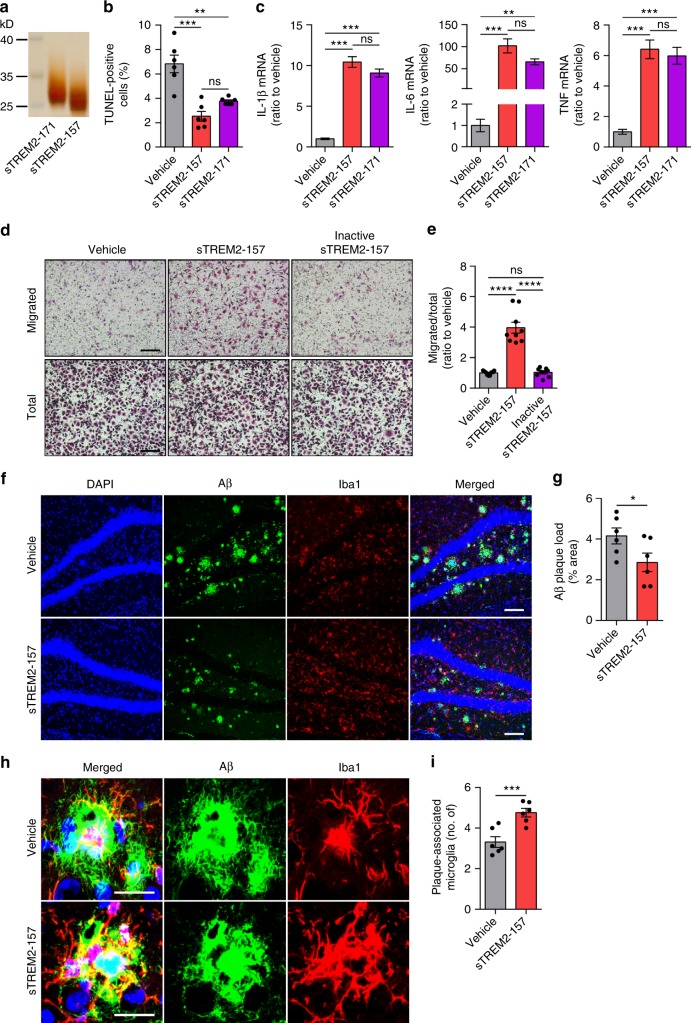
The physiological form of sTREM2 protein modulates microglial responsiveness and decreases plaque deposition. **a** The purified sTREM2-157 and sTREM2-171 proteins were analyzed by silver staining. **b** Primary microglia were cultured with 40 nM sTREM2-157 or sTREM2-171 protein for 24 h after GM-CSF withdrawal. TUNEL staining was then performed and the number of TUNEL-positive cells were quantified (*n* = 6 from three independent experiments, one-way ANOVA). **c** Primary microglia were treated with 40 nM sTREM2-157 or sTREM2-171 protein for 4 h. The relative mRNA levels of IL-1β, IL-6, and TNF shown as bar graphs were determined by quantitative real-time PCR. β-actin was used as an internal control (*n* = 3 per group, one-way ANOVA). **d** Primary microglial cells were plated onto transwell chamber inserts. Following 24-h incubation with vehicle (PBS), native sTREM2-157 protein (100 nM), or inactive sTREM2-157 protein (100 nM), cells migrated through the membrane were imaged under a Nikon inverted microscope. Scale bar, 100 µm. **e** Quantitation of the number of migrated cells in **d** (*n* = 9 from three independent experiments, one-way ANOVA). **f** Coronal sections from 5×FAD mice injected with either sTREM2-157 protein or vehicle control were stained with DAPI (blue) for nuclei, MOAB-2 (green) for Aβ, and Iba1 (red) for microglia. Representative images of the hippocampus region are shown. Original magnification ×20; scale bar, 100 μm. **g** Quantitation of amyloid plaque deposition in **f** (*n* = 6 mice, 24 fields of each group for analysis, paired Student’s *t* test). **h** Coronal sections from sTREM2-157-injected 5×FAD mice were stained with DAPI (blue) for nuclei, MOAB-2 (green) for Aβ, and Iba1 (red) for microglia. Representative z-stack images of the hippocampus region are shown. Scale bar, 25 μm. **i** Quantitation of the number of plaque-associated microglia in **h** (*n* = 6 mice, 47 plaques of vehicle and 40 plaques of sTREM2 for analysis, paired Student’s *t* test). Plaques with 50 μm in diameter were selected for analysis. All data are presented as mean ± SEM. *p < 0.05; **p < 0.01; ***p < 0.001; ****p < 0.0001; ns, not significant

At the time we started this project, the precise TREM2 cleavage site to generate sTREM2 was unknown. More recently, it was reported that TREM2 undergoes regulated shedding at the H157–S158 peptide bond, resulting in the liberation of soluble TREM2 ending at position 157 (sTREM2-157)^35–37^. Hence, we went on to test whether the physiological form of sTREM2-157 protein recapitulates the functions that we defined for the sTREM2-171 mimics (Fig. 5a). Consistent with our previous findings^43^, the sTREM2-157 protein was as efficient as the sTREM-171 in both suppressing microglial apoptosis and stimulating the production of inflammatory cytokines (Fig. 5b, c). Furthermore, the native sTREM2-157 protein but not its heat-inactivated form significantly stimulated the migration of microglial cells, consistent with the role of sTREM2-171 protein as a chemoattractant for microglia (Fig. 5d, e). Importantly, the sTREM2-157 protein largely and significantly decreased the deposition of amyloid plaques and promoted the clustering of microglia in the vicinity of plaques (Fig. 5f–i). However, sTREM2 lost these protective functions upon heat inactivation, indicating that the native protein structure is required for sTREM2 function (Supplementary Fig. 5). We therefore conclude that the physiological form of sTREM2 protein functions as efficiently as the sTREM2-171 in modulating microglial responsiveness and decreasing plaque deposition in 5×FAD mice.

### Long-term expression of sTREM2 reduces Aβ pathology

**Fig. 6:**
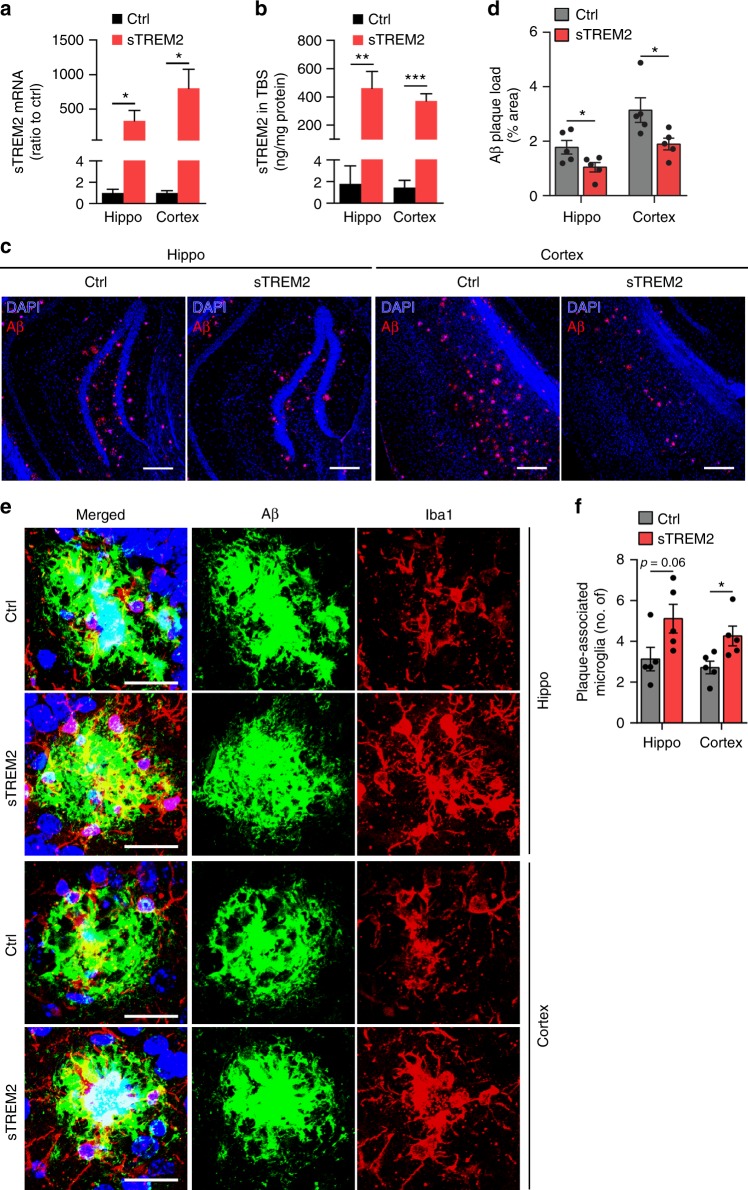
AAV-mediated sTREM2 expression reduces amyloid plaque deposition and increases the number of plaque-associated microglia. **a**, **b** AAV-mediated sTREM2 expression in 5×FAD mice. Neonatal 5×FAD mice were injected with either AAV2/8-CAG-EGFP (Ctrl) or AAV2/8-CAG-EGFP-2A-sTREM2-3×FLAG (human TREM2 1–171 aa) (sTREM2) in the cerebral ventricles and analyzed after 7 months for sTREM2 expression at the mRNA (**a**, *n* = 8 mice per group, unpaired Student’s *t* test) or protein (**b**, *n* = 8 mice for Ctrl, *n* = 9 mice for sTREM2, unpaired Student’s *t* test) levels in the cortex and hippcampus using RT-qPCR or ELISA, respectively. **c** The 5×FAD mice injected with control AAV or AAV-sTREM2 were analyzed for amyloid plaque pathology using an anti-Aβ antibody MOAB-2. Representative images of the hippocampus and the cortex of those mice are shown. Original magnification ×10; scale bar, 200 μm. Blue, DAPI; red, Aβ antibody MOAB-2. **d** Quantitation of Aβ plaque area in the hippocampus and cortex of 5×FAD mice injected with either control AAV or AAV-sTREM2 (*n* = 5 mice per group for the hippocampus or the cortex, 12 fields of the hippo and 28 fields of the cortex for analysis, unpaired Student’s *t* test). **e** Coronal sections from the 5×FAD mice injected with control AAV or AAV-sTREM2 were stained with DAPI (blue) for nuclei, MOAB-2 (green) for an amyloid plaque, and Iba1 (red) for microglia. Representative z-stack images of the hippocampus and cortex region are shown. Scale bar, 25 μm. **f** Quantitation of the number of plaque-associated microglia in **e** (*n* = 5 mice, 35 plaques of ctrl and 37 plaques of sTREM2 in the hippocampus or 64 plaques of ctrl and 67 plaques of sTREM2 in the cortex for analysis, unpaired Student’s *t* test). Plaques with 50 μm in diameter were selected for analysis. All data are presented as mean ± SEM. *p < 0.05; **p < 0.01; ***p < 0.001

To validate the findings from recombinant sTREM2 protein and to further assess the long-term impact of sTREM2 on AD-related pathology, we expressed sTREM2 in the brain of 5×FAD mice using AAV-mediated expression approach. Viruses carrying cDNAs encoding EGFP alone as a control or EGFP-2A-sTREM2-3×FLAG (spanning N-terminal amino acids 1–171 of TREM2) were injected into the cerebral ventricles of P0 neonatal 5×FAD mice. Seven months after viral injection, we found similar distribution patterns of EGFP expression in control and sTREM2-injected brains, including the cortex and hippocampus (Supplementary Fig. 6a). By analyzing potential colocalization of sTREM2 with cell-type-specific markers for astrocytes, microglia, and neurons, we found that the sTREM2 signal was colocalized primarily with NeuN-positive neurons, but not with GFAP-positive astrocytes or Iba1-positive microglia (Supplementary Fig. 6b). The absence of AAV infection to microglial cells is consistent with the previous observation that microglia are refractory to AAV^49^. Transduction of primary neurons with the sTREM2 expression construct showed that sTREM2 was efficiently secreted into the media (Supplementary Fig. 6c, d). Thus, sTREM2 possesses the tendency to contact any cell type in the brain, including microglia, after releasing into the extracellular space. The mRNA levels of sTREM2 in the cerebral cortex and hippocampus were significantly increased in 5×FAD mice, as compared with the EGFP control (Fig. 6a). This increase was further confirmed at the protein level using ELISA to measure the levels of sTREM2 in the TBS-soluble fractions (Fig. 6b).

We next evaluated the effects of sTREM2 expression on Aβ levels and plaque deposition. We found that sTREM2 significantly decreased the total plaque burden in both the hippocampus and the cortex of sTREM2-expressing 5×FAD mice relative to controls (Fig. 6c, d). Congo red-positive dense core compact plaques were also reduced in the presence of AAV-mediated sTREM2 expression (Supplementary Fig. 7a, b). To further analyze how sTREM2 affects the dynamic pools of Aβ, we examined Aβ levels in sequentially extracted tris-buffered saline (TBS)-soluble, detergent-soluble (TBSX), and detergent-insoluble (guanidine-HCl, GDN) fractions from cortical and hippocampal mouse brain tissues by ELISA^50^. The majority of Aβ in these mice at 7 months of age was fractioned in the detergent-insoluble fractions, reflecting its deposition in the amyloid plaque (Supplementary Fig. 7c, d). Consistent with decreased Aβ deposition, the concentrations of insoluble Aβ42 in the guanidine fractions were significantly lower in the cortex and hippocampus of sTREM2-expressing 5×FAD mice than the control. There were no changes in Aβ levels in TBS-soluble and TBSX-soluble fractions. Furthermore, AAV-mediated sTREM2 expression had no significant effects on the levels of APP, its cleavage products, or Aβ-degrading enzymes, consistent with the effects that we observed using a recombinant sTREM2 protein (Supplementary Fig. 7e–g). In the presence of sTREM2 expression, the number of plaque-associated microglia was significantly increased in both the hippocampus and the cortex of 5×FAD mice (Fig. 6e, f). Taken together, AAV-mediated sTREM2 expression ameliorates plaque deposition and enhances microglial enrichment in the vicinity of an amyloid plaque.

### AAV-mediated sTREM2 expression rescues behavioral deficits

**Fig. 7:**
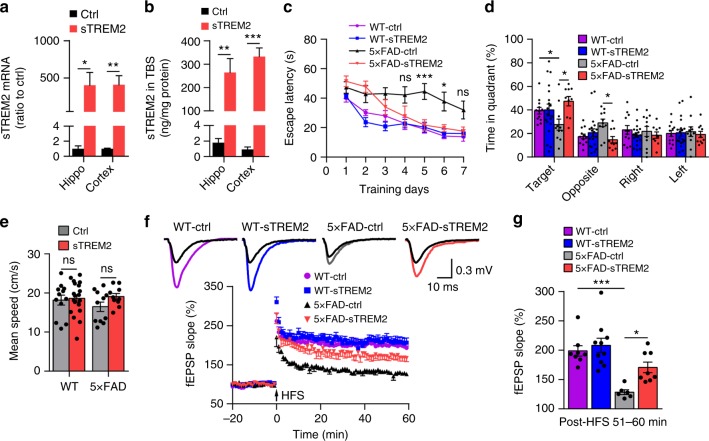
AAV-mediated sTREM2 expression rescues behavioral and LTP deficits in 5×FAD mice. **a**, **b** Neonatal WT mice were injected with either control AAV or AAV-sTREM2 in the cerebral ventricles and analyzed after 7 months for sTREM2 expression at the mRNA (**a**, *n* = 6 mice for Ctrl in hippo or cortex, *n* = 6 mice for sTREM2 in hippo and *n* = 5 mice for sTREM2 in the cortex, unpaired Student’s *t* test) or protein (**b**, *n* = 6 mice per group, unpaired Student’s *t* test) levels in the cortex and hippcampus using RT-qPCR or ELISA, respectively. **c**–**e** Morris water maze tests were performed in wild-type (WT) or 5×FAD mice 6 months after injection of either control AAV or AAV-sTREM2 (*n* = 12 mice for WT-Ctrl, *n* = 19 mice for WT-sTREM2, *n* = 11 mice for 5×FAD-Ctrl, and *n* = 10 mice for 5×FAD-sTREM2, two-way ANOVA, Bonferonni post hoc analyses). The escape latency time to reach the hidden platform was recorded during the 7-day training (**c**). Probe trial was performed 24 h after the last trial of a hidden platform task and the percentage of search time for each quadrant was recorded (**d**). Swimming speed of each group was recorded during the probe trial (**e**). **f** Time course of fEPSP measures recorded in the hippocampal CA1 region before and after 100-Hz stimulation in the Schaffer collateral region in slices from WT or 5×FAD mice 7 months after receiving control AAV or AAV-sTREM2. Normalized fEPSP slopes were plotted every 1 min for each group. **g** The averaged fEPSPs recorded 50–60 min after induction of LTP (*n* = 8 slices for WT-Ctrl, *n* = 10 slices for WT-sTREM2, *n* = 6 slices for 5×FAD-Ctrl, and *n* = 8 slices for 5×FAD-sTREM2, at least three mice per group, two-way ANOVA, Bonferonni post hoc analyses). All data are presented as mean ± SEM. *p < 0.05; **p < 0.01; ***p < 0.001; ns, not significant

We further evaluated if the beneficial effects of sTREM2 expression in reducing amyloid plaque deposition are associated with improved synaptic plasticity and cognitive function of 5×FAD mice. To analyze the potential impacts of sTREM2 expression on AD-related behavioral deficits, the spatial learning and memory function of experimental mice were evaluated by the Morris water maze 6 months after viral injection. Similar to the 5×FAD mice, the mRNA and protein levels of sTREM2 in the cerebral cortex and the hippocampus detected 7 months after AAV injection were significantly increased in WT mice (Fig. 7a, b). The 5×FAD mice were less efficient at finding the hidden platform than WT mice, indicating a deficit in spatial learning ability at 6 months of age (Fig. 7c). Importantly, sTREM2 expression rescued this spatial learning deficit, as evidenced by a reduced latency to reach the platform over the 7 days of training. During the probe trials conducted 24 h after the last training, the 5×FAD mice receiving sTREM2 viruses spent significantly more time in the target quadrant than those receiving control viruses (Fig. 7d). However, expression of sTREM2 did not affect the spatial memory of WT mice in either the hidden platform tests or probe trials (Fig. 7c, d). As controls, swim speeds were comparable in all groups of animals, thus excluding impairments in motor function (Fig. 7e). These data indicate that sTREM2 expression could rescue the spatial memory impairments in 5×FAD mice.

We next examined whether sTREM2-mediated memory improvement in 5×FAD mice was reflected at the synaptic level. We thus studied the hippocampal synaptic plasticity in the same set of animals as used for the behavioral study. Consistent with previous studies, the 5×FAD mice showed significantly impaired LTP as compared with WT mice (Fig. 7f, g)^51^. Importantly, the magnitude of LTP measured during the last 10 min post induction (51–60 min) was significantly higher in sTREM2-expressing 5×FAD mice relative to control mice (Fig. 7g). However, sTREM2 expression had a minimal effect on hippocampal LTP in WT mice. Thus, AAV-mediated expression of sTREM2 largely and specifically ameliorated LTP deficits in 5×FAD mice. Taken together, our study reveals that sTREM2 expression could rescue or ameliorate key Aβ/amyloid-induced AD phenotypes, including defective synaptic plasticity and impaired spatial memory.

## Discussion

Although it has been widely acknowledged that the CSF levels of sTREM2 are elevated in AD, it remains unknown how sTREM2 impacts the amyloid pathology and microglial behaviors in the disease context. In the present study, we demonstrated for the first time that sTREM2 is capable of reducing amyloid deposition, ameliorating synaptic impairments and memory deficits in the 5×FAD mouse model. Intriguingly, these protective effects of sTREM2 were attenuated by the depletion of microglia, suggesting a crucial role for microglia in mediating sTREM2 function. Indeed, sTREM2 enhances a variety of microglial activities known to control amyloid pathology, including microgliosis, migration, clustering in the vicinity of an amyloid plaque, as well as uptake and degradation of Aβ. As the accumulation and deposition of Aβ in the brain likely drives the pathogenic cascades of AD^4,52,53^, our study provides a critical link between Aβ pathology and the emerging role of microglia-mediated innate immune response in the pathogenesis of AD^54,55^.

Recent studies have begun to unravel the specific roles of TREM2 in modulating microglial functions. For example, the deficiency of TREM2 has been shown to decrease the number of plaque-associated microglia, reduce plaque compaction, and axonal dystrophy^11,56^. The TREM2 signaling pathways also play key roles in regulating Aβ uptake by microglial cells; loss of TREM2 results in reduced phagocytosis, while increased TREM2 gene dosage enhances phagocytic activity^11,57^. Intriguingly, the above-mentioned functions as well as the effects on microglial proliferation and migration by full-length TREM2 are well recapitulated by sTREM2. Since the soluble version of another TREM family member TREM1 was found to negatively modulate TREM1 signaling, initially, sTREM2 was postulated to similarly compete with its membrane-bound form to block TREM2 function. Nevertheless, our data suggest that sTREM2 possesses important biological and pathological roles other than acting as a decoy receptor opposing full-length TREM2 signaling.

Although many functional aspects of sTREM2 resemble those of membrane-bound TREM2, they are not identical. The biological actions of sTREM2 do not require the presence of full-length TREM2 or its intracellular adapter DAP12^43^. Moreover, the full-length TREM2 has been found to promote the formation of a neuroprotective microglia barrier that regulates amyloid compaction and insulation, thereby reducing axonal dystrophy around amyloid deposits. Despite a clear reduction of plaque deposition and the total area of dystrophic neurites by sTREM2, it does not significantly impact the amount of dystrophic neurites per plaque and thus unlikely contributes to compacting amyloid fibrils (Fig. 1). Recently, comprehensive single-cell RNA analysis of CNS immune cells in neurodegenerative conditions has discovered an essential role of TREM2 in the activation of disease-associated microglia^58,59^. In future studies, it will be informative to perform transcriptomics analysis at the single-cell level to examine how sTREM2 modulates microglial gene expression in the diseased brain.

A recent study reported that sTREM2 released from cell membranes latches onto amyloid plaque and nearby neurons^42^, indicating a direct physical interaction between sTREM2 and amyloid plaques/neurons. In fact, we have found that an oligomeric Aβ42 specifically binds to sTREM2 with a high affinity^26,27^. In addition, sTREM2 also binds to apoE, another major component of amyloid plaques^23–25^. Hence, sTREM2 is likely present in amyloid plaques and acts as a chemoattractant for microglial recruitment to the vicinity of a plaque. Although sTREM2 has been reported to co-localize with neurons in vivo, incubation of primary neuronal cultures with sTREM2 in the absence of microglia had minimal effects on the abundance of the synaptic proteins or the amplitude and frequency of both mEPSCs and mIPSCs (Supplementary Fig. 4). However, sTREM2 significantly increased the levels of several synaptic proteins and rescued the impaired LTP when administered to the brains of 5×FAD mice (Fig. 4). Intriguingly, the protective effects of sTREM2 on amyloid load and synaptic function were attenuated by the presence of a selective CSF1R kinase inhibitor, PLX3397, which nearly eliminates microglia from the 5×FAD mice (Fig. 4). Hence, our data support that microglia are the main effector cells that mediate the protective functions of sTREM2 in the context of AD. However, the microglial surface receptor(s) for sTREM2 remain unknown. Considering the fact that TREM2 is a highly promiscuous receptor engaging a wide array of ligands, the receptor(s) for sTREM2 might also be highly redundant and needs to be systematically investigated in future studies.

Although the precise mechanism mediating the ability of sTREM2 to attenuate AD pathology remains to be determined, we speculate that significantly reduced levels of Aβ and plaque load upon sTREM2 treatment may contribute to the rescue of synaptic failure and memory deficits in the case of long-term sTREM2 exposure (AAV-mediated sTREM2 expression). However, it is unlikely that the decreased amyloid plaque is responsible for improved LTP in brain slices that were treated with sTREM2 for only 1 h. There are several previous studies that have linked microglia-related functions to synaptic plasticity. For example, Parkhurst et al. showed that microglia promote learning-induced formation of glutamatergic synapses and that this effect is mediated by microglia-released brain-derived neurotrophic factor^60^. In addition, it has been demonstrated that TNF and IL-1β released by microglial cells enhance synaptic transmission and neuronal activity^61–63^. Our current study has clearly shown that sTREM2 exerts its effects in a microglia-dependent manner. Thus, it is possible that sTREM2 positively modulates synaptic plasticity by promoting neurotrophic functions of microglia. Taken together, our data suggest that sTREM2 might exert its protective functions in AD by both amyloid-dependent and amyloid-independent pathways.

In conclusion, our data provide strong evidence that sTREM2 is crucially involved in regulating microglial dynamics to control amyloid plaque development and synaptic plasticity. Although the precise mechanistic underpinnings of the disease-modulating effects require further investigation, our findings shed new light on the roles of sTREM2 in AD, and suggest that elevation of sTREM2 represents a protective response against AD pathology.

## Methods

### Mice

All animal experiments were approved by the Animal Ethics Committee of the Xiamen University and were conducted in compliance with all relevant ethical regulations for animal testing and research. Sample sizes were adequately powered to observe the effects on the basis of past experience of animal studies^43,64^. Mice were randomly selected for further biological analysis, and the investigators were blinded to group allocation during the experiments or outcome assessments.

For AAV injection, postnatal P0 mice within each genotype (WT or 5×FAD mice) were injected with control AAV or AAV-sTREM2, and both males and females were used in this study. The behavioral tests were performed at 6 months of age. For LTP and biochemical analysis, samples were harvested from AAV-injected WT or 5×FAD mice at 7 months.

For sTREM2 protein injection, 5×FAD mice at 7 months of age were injected with control (PBS or heat-inactivated sTREM2) or native sTREM2 protein into the left and right hippocampi, respectively. Seven days after injection, mice were anesthetized and perfused with ice-cold PBS. Samples were harvested for biochemical or histological analysis. Both males and females were used in this study.

To pharmacologically ablate microglia in the brain, 6-month-old (for LTP) or 7-month-old (for amyloid plaque) 5×FAD mice were fed with PLX3397 (Selleck, S7818, 290 mg/kg formulated in standard chow)^47,48^. Age-matched control groups were fed with the same standard chow but without PLX3397.

### Injection of AAV

The AAV2/8 viruses expressing human EGFP-2A-sTREM2-3×FLAG (1–171aa) (AAV-sTREM2) or EGFP (control AAV) under the control of the CAG promoter were generated by Obio Technology (Shanghai). Neonatal mice were cryoanesthetized on ice for 3 min before AAV injection. Following cessation of movement, viruses were injected into the lateral ventricles of both cerebral hemispheres with 7.5 × 10^9^ total viral particles per side using a 10-μL syringe (Hamilton, 7642-01) with a 30-G needle (Hamilton, 7803-07). After both injections were complete, pups were placed on a warming pad until they regained normal color and resumed movement. All injected pups were then returned to their mothers for care and further recovery.

### Tissue preparation

Mice were anesthetized and transcardially perfused with 50 mL of ice-cold 1× PBS. Brains were removed and dissected at the midline. For biochemical analysis, the cortex and hippocampus were dissociated and immediately snap-frozen in liquid nitrogen and stored at −80 °C for extraction of protein and RNA. For immunohistochemistry analysis, brains were fixed in 4% paraformaldehyde (PFA, Sigma-Aldrich, P1468) overnight at 4 °C, followed by transferring to 30% sucrose at 4 °C for 48 h, before being embedded for cryostat sectioning.

### Western blotting

Samples were homogenized and incubated in RIPA Lysis and Extraction Buffer (Thermo Fisher Scientific, 89900), supplemented with Protease and Phosphatase Inhibitor Cocktail (Thermo Fisher Scientific, 78440). Protein concentrations were determined using the BCA Protein Assay Kit (Thermo Fisher Scientific, 23225) according to the manufacturer’s instruction. Equal amounts of total proteins were resolved by sodium dodecyl sulfate–polyacrylamide gel electrophoresis and transferred to PVDF membranes (Millipore, IPVH00010). After blocking, the membranes were blotted by a primary antibody and detected with horseradish peroxidase-conjugated secondary antibody. Proteins were visualized using ECL Western blotting detection reagents (Millipore, WBKLS0500). Immunoreactive bands were quantified using ImageJ software. The following antibodies were used: anti-Iba1 (Wako, 016-20001, 1:500), anti-GFAP (Cell Signaling Technology, 3670S, 1:1000), anti-NR2A (Millipore, 07-632), anti-NR2B (DB Biosciences, 610416, 1:1000), anti-NR1 (Cell Signaling Technology, 5704S, 1:1000), anti-GluR1 (Millipore, MAB2263, 1:1000), anti-GluR2 (Millipore, AB1768-I, 1:1000), anti-PSD95 (Cell Signaling Technology, 3450S, 1:2000), anti-vGluT1 (Millipore, MAB5502, 1:2000), anti-Synaptophysin (Sigma-Aldrich, S5768, 1:2000), anti-APP C-terminal (369^65^, in-house, 1:1000), anti-TREM2 (R&D Systems, AF1828, 1:500), and anti-α-Tubulin (Millipore, MABT205, 1:3000).

### RNA isolation and real-time quantitative PCR analysis

Total RNA was isolated from the cortex or the hippocampus using TRIzol reagent (Thermo Fisher Scientific, 15596018). One microgram of RNA was reverse transcribed into the first-strand cDNA using cDNA Synthesis SuperMix (TransGen Biotech, China, AT314-02) according to the manufacturer’s protocol. Quantitative PCR was performed using the FastStart Universal SYBR Green Master mix (Roche, 04913914001). The real-time PCR was performed on 7500 fast (ABI) or 480 LightCycler (Roche). The primer sequences were as follows: Iba1-Forward: GTCCTTGAAGCGAATGCTGG, Iba1-Reverse: CATTCTCAAGATGGCAGATC; GFAP-Forward: TCCTGGAACAGCAAAACAAG, GFAP-Reverse: CAGCCTCAGGTTGGTTTCAT; IL-1β-Forward: GCAACTGTTCCTGAACTCAACT, IL-1β-Reverse: ATCTTTTGGGGTCCGTCAACT; IL-6-Forward: CAATGGCAATTCTGATTGTATG, IL-6-Reverse: AGGACTCTGGCTTTGTCTTTC; TNF-Forward: CCCTCACACTCAGATCATCTTCT, TNF-Reverse: GCTACGACGTGGGCTACAG; sTREM2-Forward: AACTTGTGGCTGCTGTCCTT, sTREM2-Reverse: GGTAGAGACCCGCATCATGG; IDE-Forward: AATCCGGCCATCCAGAGAATA, IDE-Reverse: GGGTCTGACAGTGAACCTATGT; NEP-Forward: CTCTCTGTGCTTGTCTTGCTC, NEP-Reverse: GACGTTGCGTTTCAACCAGC; ECE1-Forward: CAGGTGGTCACAGCTCACTAC, ECE1-Reverse: GGTATCCAGTCAGGACCTTTTCA; MMP2-Forward: CAAGTTCCCCGGCGATGTC, MMP2-Reverse: TTCTGGTCAAGGTCACCTGTC; MMP9-Forward: GCAGAGGCATACTTGTACC, MMP9-Reverse: TGATGTTATGATGGTCCCACTTG.

### Microglial migration in transwell assays

Mixed glial cultures were isolated from WT mice at postnatal day 1–2 and were plated onto poly-l-lysine-coated flasks and grown in DMEM supplemented with 10% fetal bovine serum (FBS) (Gibco). After 3 days, the medium was changed to that which contained 25 ng/ml granulocyte-macrophage CSF (GM-CSF) and 10% FBS. Primary microglial cells were harvested by shaking after 10–12 days in culture and once every 3 days thereafter (up to four harvests). Microglial migration assay was performed in transwell cell culture inserts with a 8-μm pore (Costar, 3422). Primary microglial cells (10^5^) suspended in 100 μL of serum-free DMEM were added to the upper chamber of the inserts with 600 μL of serum-free DMEM in the bottom chamber. After 1-h incubation at 37 °C and 5% CO_2_, the bottom medium was replaced with DMEM containing PBS (vehicle), 100 nM native sTREM2, or heat-inactivated sTREM2 and the culture continued for 24 h. For analysis of the migrated cell number, cells remaining on the upper surface of the membrane were removed by scraping with a cotton swab and the inserts were washed three times with PBS. Cells were then fixed with 4% PFA for 20 min, followed by staining with hematoxylin and eosin. The total number of cells on the membrane without scraping was also counted in separate wells and the quantification for the microglial migration was normalized to the total final number of cells.

### ELISA analyses

For Aβ ELISA, the cortex or the hippocampus was sequentially extracted with TBS, TBSX, and guanidine-HCl (GDN)^50^. The levels of Aβ40 or Aβ42 were measured using sandwich ELISA techniques with monoclonal antibody (mAb) 2.1.3 (human Aβx-42 specific, 20 μg/mL, in-house) and mAb 13.1.1 (human Aβx-40 specific, 20 μg/mL, in-house) for capture and horseradish peroxidase (HRP)-conjugated mAb Ab5 (human Aβ1-16 specific, 1 μg/mL, in-house) for detection^64^. The ELISA was developed using ELISA TMB (Sigma-Aldrich, T8665). Synthetic human Aβ40 (AnaSpec, AS-24236) or Aβ42 peptide (AnaSpec, AS-20276) was used to generate the standard curves for each assay. Human or mouse sTREM2 ELISA assay was established in-house. A 96-well plate was coated with a TREM2 antibody (R&D Systems, MAB17291-100, 1:1000) in coating buffer (0.05 M Carbonate buffer, pH 9.6) overnight at 4 °C. The plate was then blocked in blocking buffer (1% Block Ace in PBS) for 4 h at room temperature (RT), subsequently washed three times with PBS, and incubated with samples diluted 1:5 with assay buffer (0.2% bovine serum albumin and 0.05% Tween 20 in PBS) overnight at 4 °C. Plates were washed five times with wash buffer (0.05% Tween 20 in PBS) before incubation for 2 h at RT with human TREM2 biotinylated antibody (R&D Systems, BAF1828, 1:3000) or mouse Trem2 biotinylated antibody (R&D Systems, BAF1729, 1:3000). After the washing steps, the plates were incubated with Streptavidin Poly-HRP40 Conjugate (Fitzgerald, 65R-S104PHRP, 1:3000) for 1 h in the dark. After five additional washing steps, the plates were developed by adding the TMB substrate (Sigma-Aldrich, T5569) and read at 620 nm on a Varioskan Flash Multimode Reader (Thermo Fisher Scientific).

### Immunohistochemistry and microscopy

Mouse coronal sections were cut (12-µm thick) with a cryostat and mounted on poly-l-lysine precoated glass slides, air-dried overnight at 37 °C, and subsequently processed for staining. The sections were washed in 1× PBS for 15 min and incubated with blocking buffer (PBS with 5% normal donkey serum and 0.2% Triton X-100) for 1 h at RT, followed by incubation with primary antibodies for 48 h at 4 °C and the Alexa-fluorophore-conjugated secondary antibodies (Thermo Fisher Scientific, A-21203, A-21202, A-21207, A-21244, or A-21247, 1:400) for 2 h at RT in the dark. Sections were washed and sealed with an anti-fade reagent (Life Technologies, P36935). For confocal microscopy, a NIKON A1R Plus confocal microscope was used to acquire all images; laser and detector settings were maintained constant in the same experiment. For analysis of the amyloid plaque-associated microglia, 6-μm z-stacks (consisting of 13 optical slices of 0.5-μm thickness) were acquired and the maximum intensity projections were generated. To examine the colocalization of an amyloid plaque and CD68, 6-μm z-stacks (consisting of 13 optical slices of 0.5-μm thickness) were acquired and 3D reconstruction was conducted using the Imaris software (Bitplane). The following antibodies of immunohistochemistry detection were used in this study: anti-Iba1 (Wako, 019-19741, 1:200), anti-Aβ (MOAB-2, Abcam, ab126649, 1:400), anti-TREM2 (R&D Systems, AF1828, 1:50), anti-Lamp1 (DB Biosciences, 553792, 1:50), anti-Ki67 (ThermoFisher, 14-5698-82, 1:50), anti-CD68 (Bio-Rad, MCA1957, 1:50), anti-NeuN (R&D Systems, MAB377, 1:100), and anti-GFAP (R&D Systems, AB5804, 1:800).

### Brain stereotaxic injection

The 5×FAD mice at 7 months of age were anesthetized and placed in a stereotaxic frame, a skin incision was made, and holes were drilled at x (±2.0 mm from bregma) and y (−2.0 mm from bregma). A total of 6 μg of sTREM2 protein in 2.0 μL of PBS, or heat-inactivated sTREM2 in 2.0 μL of PBS, or 2.0 μL of PBS alone was delivered at 0.20 µL/min at z-depths of 2.0 mm to the right and left hemispheres, respectively. The syringe was left in place for 10 min after each injection before being withdrawn slowly. Seven days after injection, mice were anesthetized and perfused with ice-cold PBS. For biochemical analysis, hippocampi from the left or the right brain were dissected out, snap-frozen in liquid nitrogen, and stored at −80 °C for extraction of protein and RNA. For histologic analysis, brains were fixed in 4% PFA overnight at 4 °C and transferred to 30% sucrose for 48 h before being embedded for cryostat sectioning.

### Aβ phagocytosis assays

For the in vitro assay, primary microglial cells were seeded into 12-well culture plates at a density of 2 × 10^5^ cells per well and were cultured for 24 h after GM-CSF withdrawal. Cells were treated with 160 nM sTREM2 protein in serum-free DMEM for 12 h, followed by treatment with a phagocytosis inhibitor cytochalasin D (Calbiochem, 250255, 10 µM) for 30 min and incubation with 500 nM FAM-Aβ42 oligomer for an additional 3 h. Cells were trypsinized and washed with cold PBS for detection of FAM fluorescence using fluorescence-activated cell sorting (FACS). The in vivo phagocytosis assay was performed, as described previously with slight modifications^66^. The 7-month-old 5×FAD mice were injected with sTREM2 as described above. After 7 days, mice were injected intraperitoneally with methoxy-X04 (Toris, 4920) at 10 mg/kg in 10% DMSO/90% PBS (pH 12) for 3 h. Anesthetized mice were perfused with ice-cold PBS and the hippocampi were isolated and chopped into pieces and digested in DMEM/F12 containing 1 mg/mL Papain (Sigma, P4762), 1.2 U/mL Dispase II (Sigma, D4693), 20 U/mL DNAse I (Sigma, D5025), and 100 U/mL Collagenase IV (ThermoFisher, 17104019) at 37 °C for 30 min. The homogenization was achieved by pipetting gently up and down. The homogenates were washed and filtered through a 70-µm cell strainer. Hippocampal cells were centrifuged at 500 *g* for 5 min at 4 °C and resuspended in PBS. Cells were incubated with the Fc receptor blocking antibody CD16/CD32 (BD Bioscience, 553141, 1:100) for 10 min at 4 °C to prevent unspecific binding. Cells were washed once and then resuspended in 200 µL of PBS containing CD11b-APC (eBioscience, 17-0112-82, 1:100) and CD45-FITC (eBioscience, 11-0451-85, 1:100) and incubated for 1 h at 4 °C. Cells were washed again and resuspended in 2% FBS/PBS for FACS analysis. The frequencies of viable methoxy-X04^+^CD11b^+^CD45^low^ microglia were measured by flow cytometry using a CytoFlex S (Beckman) and analyzed using FlowJo (Tree Star). WT mice injected with methoxy-X04 were used as a control to determine the methoxy-X04 threshold for non-phagocytosing cells.

### Intracellular Aβ clearance

Primary microglia were treated with vehicle or sTREM2 protein (160 nM) in serum-free DMEM medium for 12 h, followed by treatment with lysosomal enzyme inhibitors (Pepstatin A: 10 µM, Leupeptin: 100 µM, and E-64d: 50 µM) or chloroquine (Sigma-Aldrich, C6628, 40 µM) for 30 min and incubation with a 500 nM Aβ42 oligomer for an additional 3 h. Cells were harvested with trypsin and cell pellets were washed twice with PBS. Microglia were lysed in lysis buffer (5 M guanidine in 50 mM Tris-HCl, pH 8.0). Lysates were centrifuged at 13,400 *g* at 4 °C for 15 min. Supernatants were collected and further used for quantifying the amounts of cell-associated Aβ42 using ELISA. The amounts of degraded Aβ42 were calculated by subtracting the cell-associated Aβ in the absence of inhibitors from that in the presence of inhibitors.

### Electrophysiological recording

For LTP recording, experiments were performed, as previously reported with modifications^67^. In brief, animals were deeply anesthetized with isoflurane and killed by decapitation. The brain was quickly removed and dissected in an ice-cold cutting solution containing 64 mM NaCl, 2.5 mM KCl, 1.25 mM NaH_2_PO_4_, 10 mM MgSO_4_, 0.5 mM CaCl_2_, 26 mM NaHCO_3_, 10 mM glucose, and 120 mM sucrose. Acute coronal slices (400 μm) were prepared using a Vibroslice (VT 1000 S; Leica). Slices were allowed to recover for 30 min at 34 °C and then at RT for at least 1 h before recording in the artificial CSF (aCSF) containing 126 mM NaCl, 2.5 mM KCl, 1.25 mM NaH_2_PO_4_, 2 mM MgSO_4_, 2 mM CaCl_2_, 26 mM NaHCO_3_, and 10 mM glucose. Slices were transferred to the recording chamber and superfused with aCSF (2 mL/min) saturated with 95% O_2_/5% CO_2_ (volume/volume) at 34 °C. For sTREM2 protein treatment, brain slices were incubated with 50 nM recombinant sTREM2 protein for 1 h at RT, and transferred to the chamber for LTP recording. fEPSPs were evoked every 20 s in the CA1 stratum radiatum by stimulating the SCs/commissural pathway (for CA1) with a bipolar stimulating electrode (FHC, Inc.) and recorded using a Multi-Clamp 700B amplifier (Molecular Devices) and Clampex10.5 acquisition software (Molecular Devices) and digitized with Digidata 1550 A (Molecular Devices) with glass pipettes (1–3 MΩ) filled with aCSF. Test stimuli consisted of monophasic 0.1-ms pulses of constant currents (with intensity adjusted to produce 25% of the maximum response) at a frequency of 0.05 Hz. After a 20-min-stable baseline was established, LTP was induced in the CA1 area by two trains of 100-Hz stimuli with the same intensity of the test stimulus. The strength of synaptic transmission was determined by measuring the initial (20–80% rising phase) slope of fEPSPs.

Whole-cell voltage-clamp recordings were performed on cortical neurons cultured for 12–14 DIV^68^. For sTREM2 protein treatment, neurons were incubated with 50 nM recombinant sTREM2-Fc or Fc protein prepared as previously described^43^ for 1 h, and transferred to the chamber for analysis. Recordings were conducted in a submerged recording chamber perfused (1–1.5 mL/min) with aCSF containing (in mM) 126 NaCl, 2.5 KCl, 1.2 NaH_2_PO_4_, 2.4 MgCl·6H_2_O, 1.2 CaCl_2_, 18 NaHCO_3_, and 11 Glucose, equilibrated with 95% O_2_ and 5% CO_2_ at RT. The patch pipette (5–8 MΩ) was filled with a standard intracellular solution containing (in mM) 140 CsCH_3_SO_3_, 2 MgCl·6H_2_O, 5 TEA-Cl, 10 HEPES, 2.5 MgATP, 0.3 Na_2_GTP, and 1 EGTA, pH 7.2–7.4 (with CsOH). mEPSCs and mIPSCs were recorded with 1 μM tetrodotoxin (TTX; Tocris, Cat. No. 1069) in voltage-clamp mode at a holding potential of −70 mV and 0 mV, respectively. Series resistance was monitored throughout the recording and was <30 MΩ. Data were filtered at 2 kHz and sampled at 10 kHz. Whole-cell recording was made using patch-clamp amplifiers (Multiclamp 700B). Data acquisition was performed using digitizers (DigiData 1440A). mEPSC and mIPSC were analyzed off-line with MiniAnalysis software (Synaptosoft Inc., Fort Lee, NJ).

### Morris water maze

Experiments were conducted in a circular 120-cm-diameter pool filled with opaque water kept at 21 °C. The mice were given four training trials per day for 7 consecutive days, during which the platform was left in the same position. The time taken to reach the platform (escape latency) was measured, and the average of four trials was determined. If the mouse could not find the escape platform within 60 s, it was placed on the platform for 10 s. A probe trail without a platform was performed 24 h after the last trial of the hidden platform test. Percentage search time for each quadrant was recorded for 60 s. Mice were tracked by a video camera (SONY) in both training and probe trials. Collected data were analyzed by SMART 2.5 VIDEO TRACKING software (Panlab, Harvard Apparatus).

### Statistical analysis

All the electrophysiological data and histologic data analyses were performed blindly. Statistical tests were performed using GraphPad Prism 5 or 6 (GraphPad Software, La Jolla, CA). Two-way ANOVA followed by Bonferonni post hoc analyses or unpaired Student’s *t* test was used to analyze AAV and neuron data. Paired Student’s *t* test was used to analyze sTREM2 protein-injected experiments. The statistical method for each quantitated data was described in the Figure legend. All data are shown as mean ± SEM and specific *n* values were reported in each Figure legend. Levels of significance are as follows: ^*^p < 0.05, ^**^p < 0.01, ^***^p < 0.001, ^****^p < 0.0001. *p* < 0.05 was considered as stastically significant.

### Reporting Summary

Further information on experimental design is available in the Nature Research Reporting Summary linked to this article.

## Supplementary information

Supplementary Information

Reporting Summary

Source Data

## Data Availability

The data underlying Figs. 1c, 1d, 1f, 1h, 1i, 2a, 2c, 2e, 2f, 2h, 3b, 3d, 3f, 3h, 3i, 3j, 4c, 4d, 4e, 4f, 4h, 4j, 5b, 5c, 5e, 5g, 5i, 6a, 6b, 6d, 6f, 7a, 7b, 7c, 7d, 7e, 7f, 7g, and Supplementary Figs. 1a, 2a, 2b, 3a, 3b, 3c, 3d, 3e, 4b, 4c, 4d, 4e, 4f, 4g, 4i, 4k, 4l, 4m, 5c, 5e, 6c, 6d, 7b, 7c, 7d, 7e, 7f, 7g are provided as a Source Data file. All other data that support the findings of this study are available from the corresponding author upon request.
