# Author Correction: Soluble TREM2 ameliorates pathological phenotypes by modulating microglial functions in an Alzheimer’s disease model

**DOI:** 10.1038/s41467-019-10950-2

**Published:** 2019-07-02

**Authors:** Li Zhong, Ying Xu, Rengong Zhuo, Tingting Wang, Kai Wang, Ruizhi Huang, Daxin Wang, Yue Gao, Yifei Zhu, Xuan Sheng, Kai Chen, Na Wang, Lin Zhu, Dan Can, Yuka Marten, Mitsuru Shinohara, Chia-Chen Liu, Dan Du, Hao Sun, Lei Wen, Huaxi Xu, Guojun Bu, Xiao-Fen Chen

**Affiliations:** 1 Fujian Provincial Key Laboratory of Neurodegenerative Disease and Aging Research, Institute of Neuroscience, School of Medicine, Xiamen University, Xiamen, 361102 China; 2 Department of Traditional Chinese Medicine, School of Medicine, Xiamen University, Xiamen, 361102 China; 3 Xiamen Key Laboratory of Chiral Drugs, School of Medicine, Xiamen University, Xiamen, 361102 China; 4 Shenzhen Research Institute of Xiamen University, Shenzhen, 518063 China; 5 Department of Neuroscience, Mayo Clinic, Jacksonville, FL 32224 USA; 6 School of Medicine, Xiamen University, Xiamen, 361102 China; 7 Neuroscience Initiative, Sanford-Burnham-Prebys Medical Discovery Institute, La Jolla, CA 92037 USA

**Keywords:** Alzheimer's disease, Neuroimmunology

Correction to: *Nature Communications* 10.1038/s41467-019-09118-9, published online 25 March 2019.

The original version of this Article omitted the following from the end of the Acknowledgements: ‘X.C. also received funding from the Shenzhen Basic Research Program JCYJ20170818140904167.’ This has now been corrected in both the PDF and HTML versions of the Article.

