# Supplementary material for: Soluble TREM2 ameliorates pathological phenotypes by modulating microglial functions in an Alzheimer’s disease model
Source: Nat Commun. 2019 Mar 25;10:1365. doi: 10.1038/s41467-019-09118-9 (PMC6433910; doi:10.1038/s41467-019-09118-9)

**Supplemental Information**

**Soluble TREM2 ameliorates pathological phenotypes by modulating microglial functions in an Alzheimer’s disease model**

**Zhong et al.**


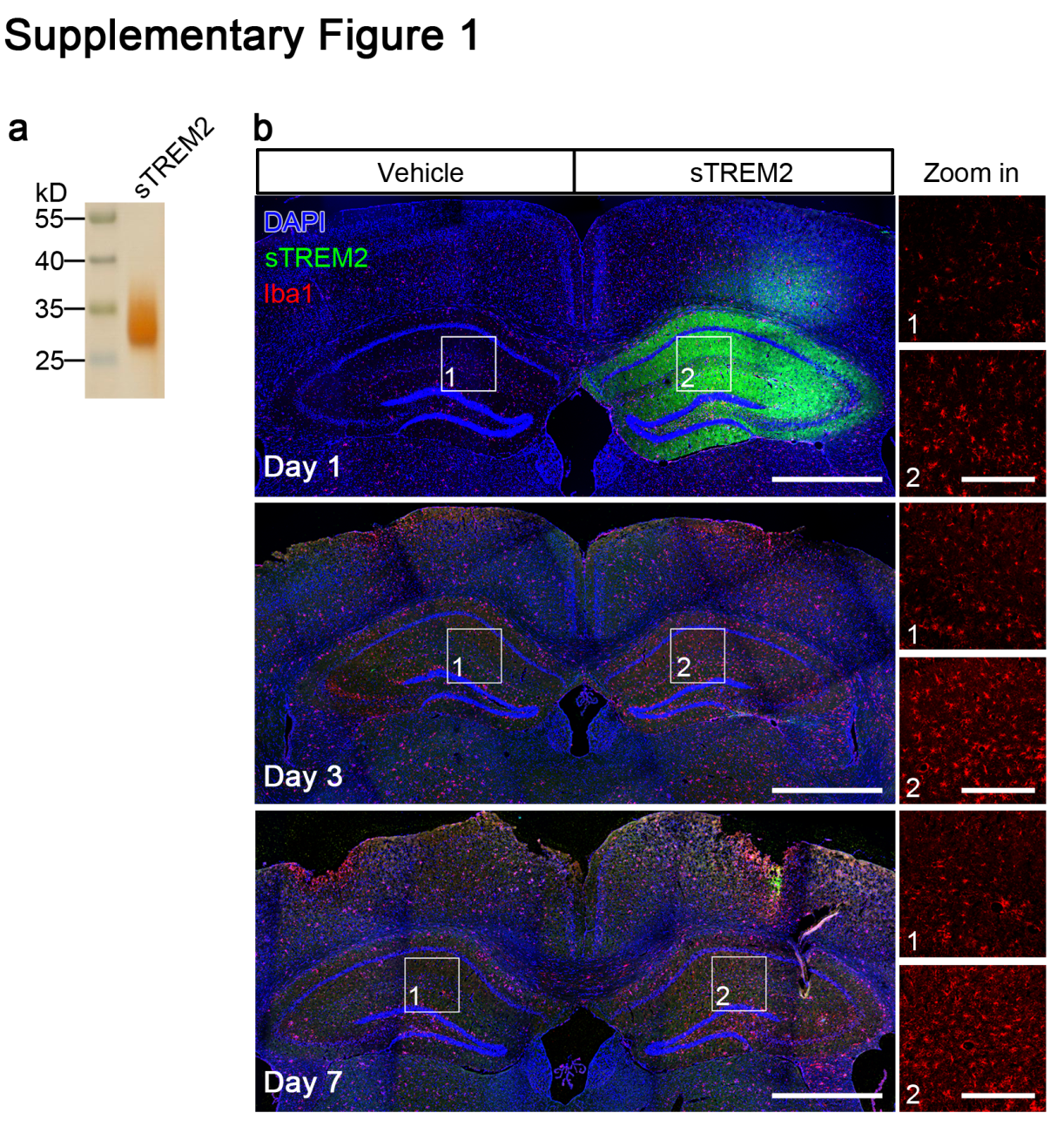


**Supplementary Figure 1. The distribution pattern of recombinant sTREM2 protein injected into the hippocampus of 5xFAD mice.**

**a** The purified sTREM2 protein was analyzed by silver staining.

**b** Coronal sections of vehicle- or sTREM2-injected 5xFAD mice on the 1^st^-, 3^rd^- and 7^th^-day postinjection were stained with DAPI (blue) for nuclei, human TREM2 (green) for sTREM2 and Iba1 (red) for microglia. Representative images of sTREM2 distribution are shown. Scale bar, 1000 μm. Zoom in images on the right with scale bar equal to 250 μm.


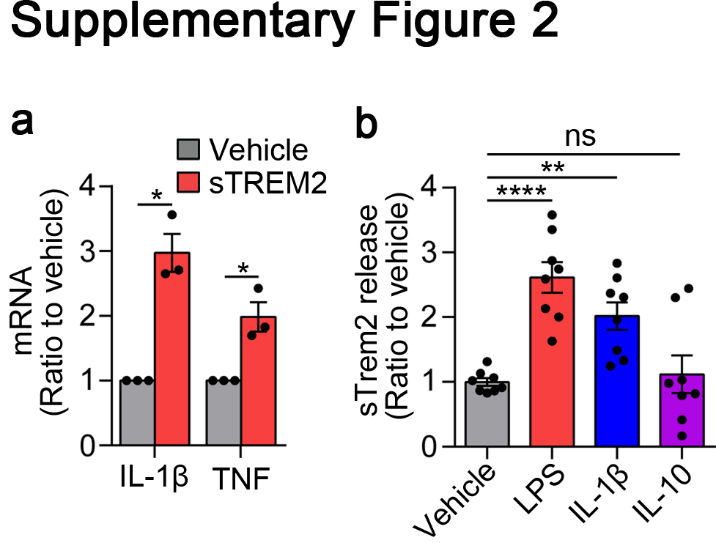


**Supplementary Figure 2. IL-1β and LPS enhance the production of sTREM2.**

**a** The hippocampus was dissected from vehicle- or sTREM2-injected 5xFAD mice. After RNA extraction, the relative mRNA levels of IL-1β and TNF in the hippocampus shown as bar graph were determined by quantitative real-time PCR. β-Actin was used as an internal control (n = 3 mice per group, paired Student’s t test).

**b** Primary microglia were cultured in serum-free DMEM for 16 hours, followed by treatment with vehicle (PBS), 200 ng/mL LPS, 100 ng/mL IL-1β or 100 ng/mL IL-10 for additional 2 hours. The levels of sTrem2 in the conditioned media were measured by ELISA (n = 8 from three independent experiments, One-Way ANOVA followed by Dunnett’s multiple comparisons test).

All data are presented as mean ± SEM. *, p<0.05; **, p<0.01; ****, p<0.0001; ns, not significant.


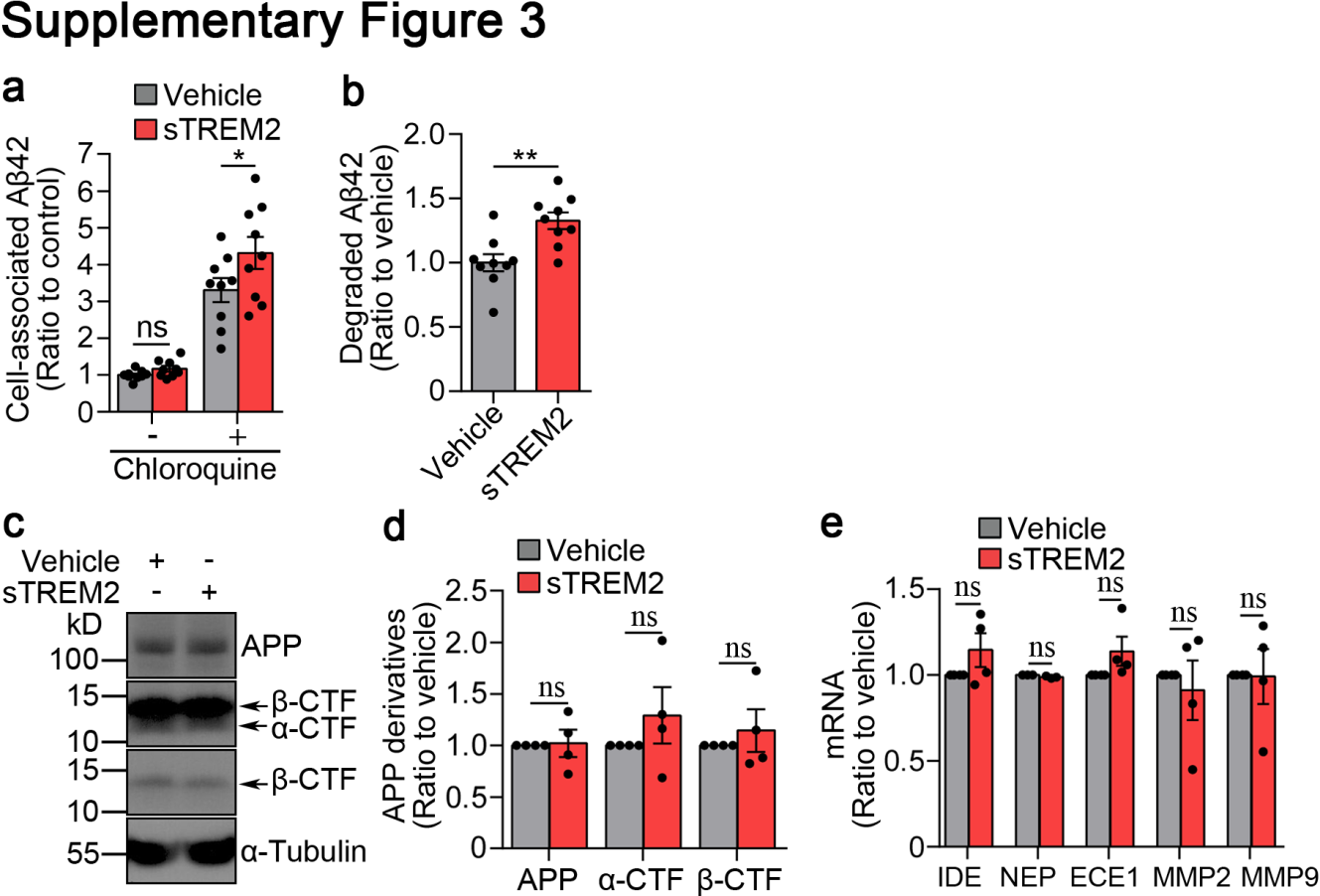


**Supplementary Figure 3. Soluble TREM2 does not affect APP processing nor the levels of Aβ degrading enzymes.**

**a** Microglia from WT mice were treated with vehicle or sTREM2 protein (160 nM) for 3 hours, followed by treatment with a lysosomal inhibitor chloroquine for 30 min and incubation with 500 nM Aβ42 oligomer for additional 3 hours. The amounts of cell-associated Aβ42 were quantified by ELISA (n = 9 from three independent experiments, two-way ANOVA, Bonferonni post hoc analyses).

**b** The amounts of degraded Aβ42 were calculated by subtracting the cell-associated Aβ in the absence of chloroquine from that in the presence of inhibitor (n = 9 from three independent experiments, unpaired Student’s t test).

**c**, **d** Western blotting analysis of APP and its derivatives, including APP, β-CTF and α-CTF, using the 369 antibody (1:1000) after sTREM2 injection into the 5xFAD mice (n = 4 per group, paired Student’s t test).

**e** The relative mRNA levels of *IDE*, *NEP*, *ECE1*, *MMP2* and *MMP9* in the hippocampus of sTREM2-injected mice shown as bar graph were determined by quantitative real-time PCR. β-Actin was used as an internal control (n = 3-4 per group, paired Student’s t test).

All data are presented as mean ± SEM. *, p<0.05; **, p<0.01; ns, not significant.


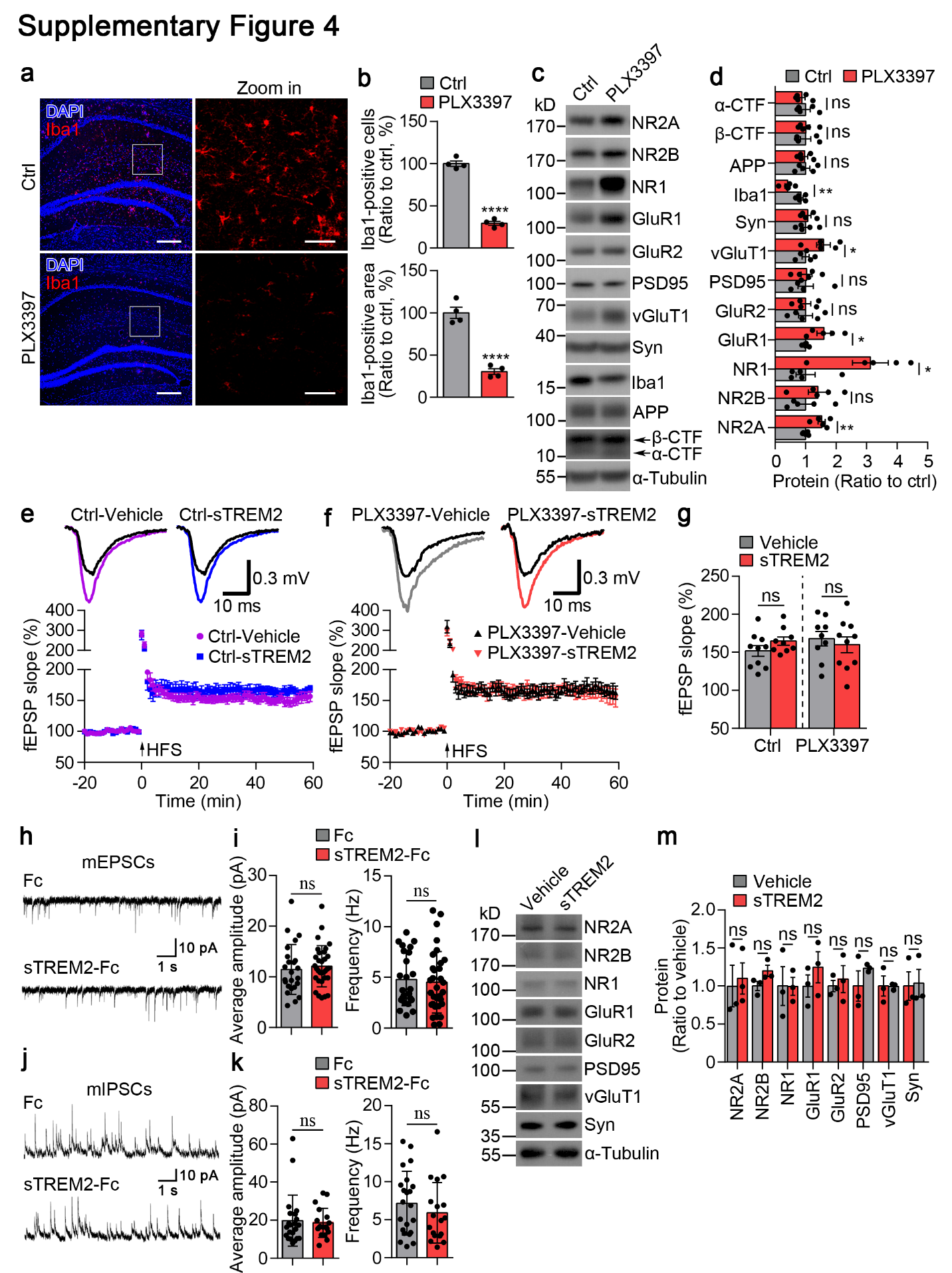


**Supplementary Figure 4. Soluble TREM2 does not directly modulate neuronal function.**

**a** Coronal sections from control chow or PLX3397-fed 5xFAD mice were stained with DAPI (blue) for nuclei and Iba1 (red) for microglia. Representative images of the hippocampus regions are shown. Original magniﬁcation 10x; scale bar, 200 μm. Zoom in images on the right with scale bar equal to 50 μm.

**b** Quantitation of the number or the area of microglia in **a** (n = 4 mice, 12-14 fields of each group for analysis, unpaired Student’s t test).

**c** The protein levels of NR2A, NR2B, NR1, GluR1, GluR2, PSD95, vGluT1, Synaptophysin (Syn), Iba1, APP, β-CTF and α-CTF in the hippocampi of the above-mentioned mice were analyzed by Western blotting.

**d** Quantitation of the protein levels in **c** (n = 5 mice per group, unpaired Student’s t test).

**e**, **f** Brain slices from control chow (e) or PLX3397-fed (f) WT mice were incubated with 50 nM recombinant sTREM2 protein for 1 hour at room temperature, and transferred to the chamber for LTP recording. Time course of fEPSP measures were recorded in the hippocampal CA1 region before and after 100 Hz stimulation in the Schaffer collateral region. Normalized fEPSP slopes were plotted every 1 min for each group.

**g** The averaged fEPSPs recorded 50-60 min after induction of LTP (n = 9-10 slices from four mice per group, unpaired Student’s t test).

**h** Representative traces of whole-cell patch-clamp miniature excitatory postsynaptic currents (mEPSCs) recordings from primary neurons (DIV 12-14) treated with 50 nM Fc or sTREM2-Fc for 1 hour.

**i** Average amplitude and frequency of mEPSCs from Fc- or sTREM2-Fc-treated neurons (n = 24 neurons for Fc; n = 32 neurons for sTREM2-Fc. Unpaired Student’s t test).

**j** Representative traces of miniature inhibitory synaptic currents (mIPSCs) recordings from primary neurons (DIV 12-14) treated with 50 nM Fc or sTREM2-Fc for 1 hour.

**k** Average amplitude and frequency of mIPSCs from Fc- or sTREM2-Fc-treated neurons (n = 22 neurons for Fc; n = 18 neurons for sTREM2-Fc. Unpaired Student’s t test).

**l** Primary neurons (DIV 12) were treated with vehicle or 50 nM sTREM2 for 24 hours. The cell lysates were harvested and analyzed with Western blotting for synaptic proteins.

**m** Quantitation of Western blots in **l** (n = 3 per group, unpaired Student’s t test).

All data are presented as mean ± SEM. *, p<0.05; **, p<0.01; ****, p<0.0001; ns, not significant.


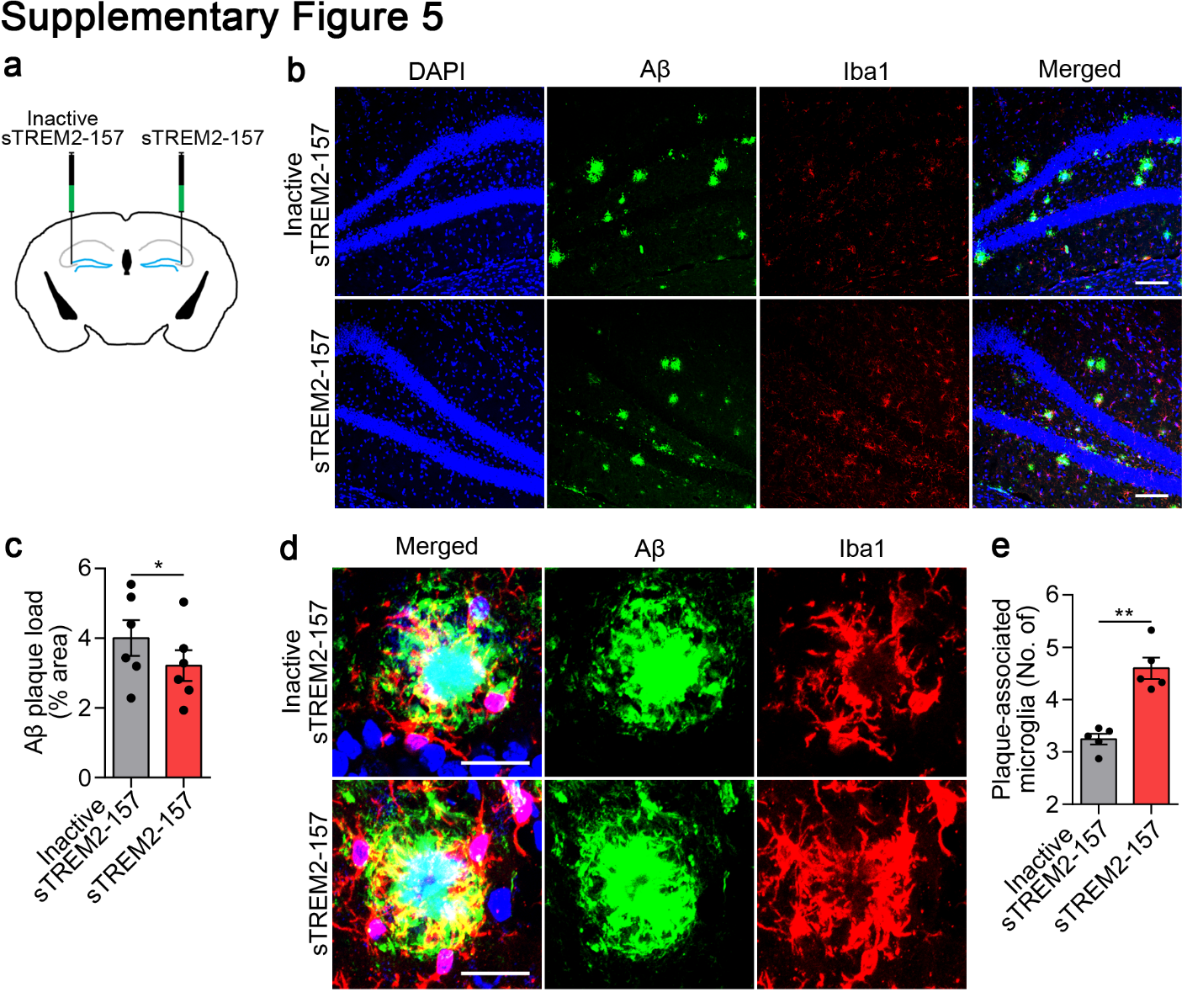


**Supplementary Figure 5. Soluble TREM2 decreases plaque deposition and increases plaque-associated microglia.**

**a** The 5xFAD mice at 7-month old age were injected with 6 μg heat-inactivated sTREM2-157 in the left hemisphere and native sTREM2 protein in the right hemisphere.

**b** The 5xFAD mice were injected with either native sTREM2-157 or heat-inactivated sTREM2-157 protein. After 7 days, coronal sections were stained with DAPI (blue) for nuclei, MOAB-2 (green) for Aβ and Iba1 (red) for microglia. Representative images of hippocampus region are shown. Original magniﬁcation 20x; scale bar, 100 μm.

**c** Quantitation of amyloid plaque deposition in **b** (n = 6 mice, 23 fields of each group for analysis, paired Student’s t test).

**d** Coronal sections from **a** were stained with DAPI (blue) for nuclei, MOAB-2 (green) for Aβ and Iba1 (red) for microglia. Representative z stack images of the hippocampus region are shown. Scale bar, 25 μm.

**e** Quantitation of the number of plaque-associated microglia in **d** (n = 5 mice, 44 plaques of vehicle and 37 plaques of sTREM2 for analysis, paired Student’s t test). Plaques with 50 μm in diameter were selected for analysis.

All data are presented as mean ± SEM. *, p<0.05; **, p<0.01.

**
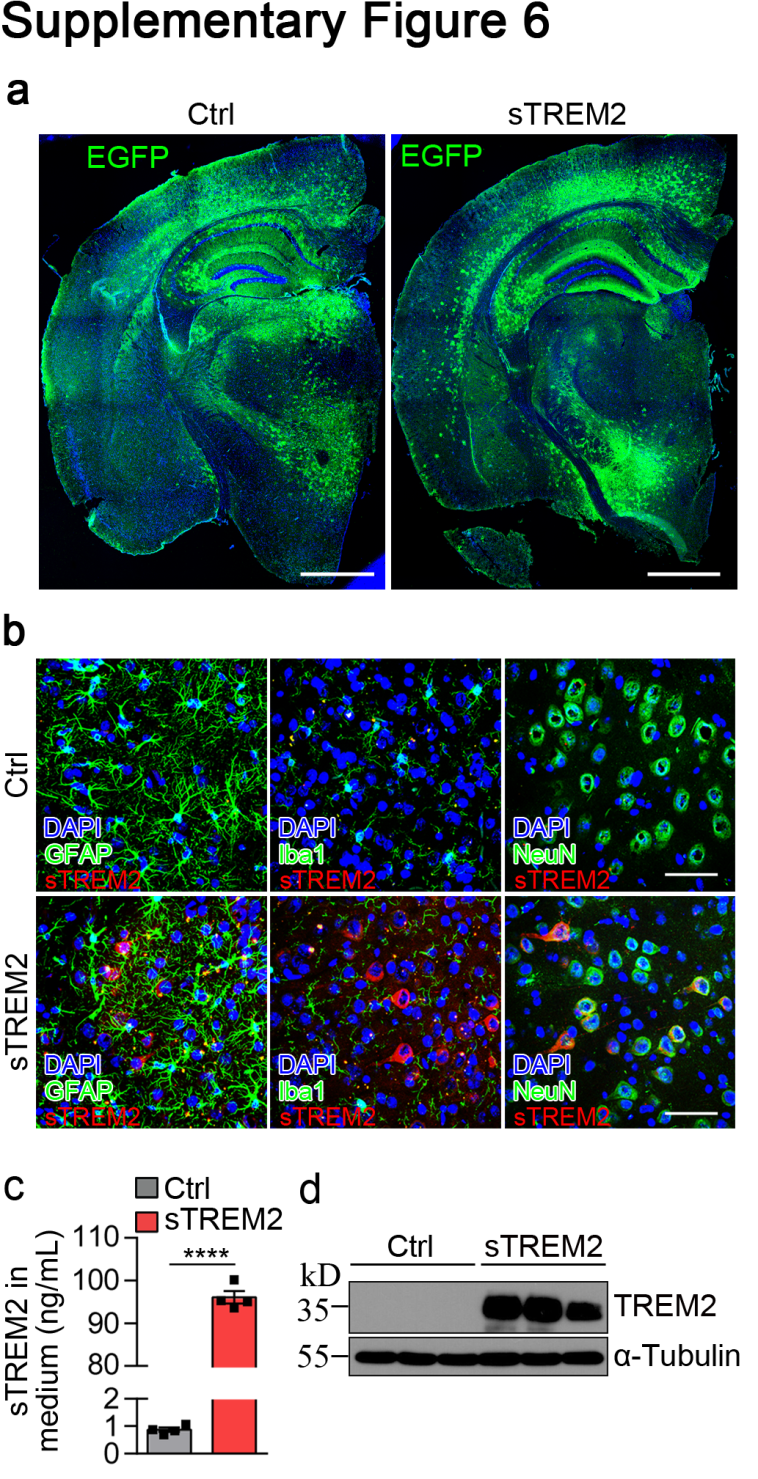
Supplementary Figure 6. The distribution pattern of AAV-mediated sTREM2 expression in 5xFAD mice.**

**a** The 5xFAD mice received either control AAV (Ctrl) or AAV-sTREM2 (sTREM2) were stained by EGFP for analysis of AAV distribution. Representative images of control or sTREM2-injected brain slices are shown. Scale bar, 1000 μm. Blue, DAPI; Green, EGFP.

**b** The 5xFAD mice received either control AAV (Ctrl) or AAV-sTREM2 (sTREM2) were co-immunostained with human TREM2 antibody for sTREM2 expression and antibodies for cell type-specific markers (GFAP: astrocyte; Iba1: microglia; NeuN: neuron). Scale bar, 50 μm.

**c-d** Primary neurons at DIV 3 were infected with either control AAV (Ctrl) or AAV-sTREM2 (sTREM2). Twelve days postinfection, the levels of sTREM2 in the medium or lysate were measured using human TREM2 ELISA assay (c) or Western blotting, respectively (d) (n = 4, unpaired Student’s t test).

All data are presented as mean ± SEM. ****, p<0.0001.

**
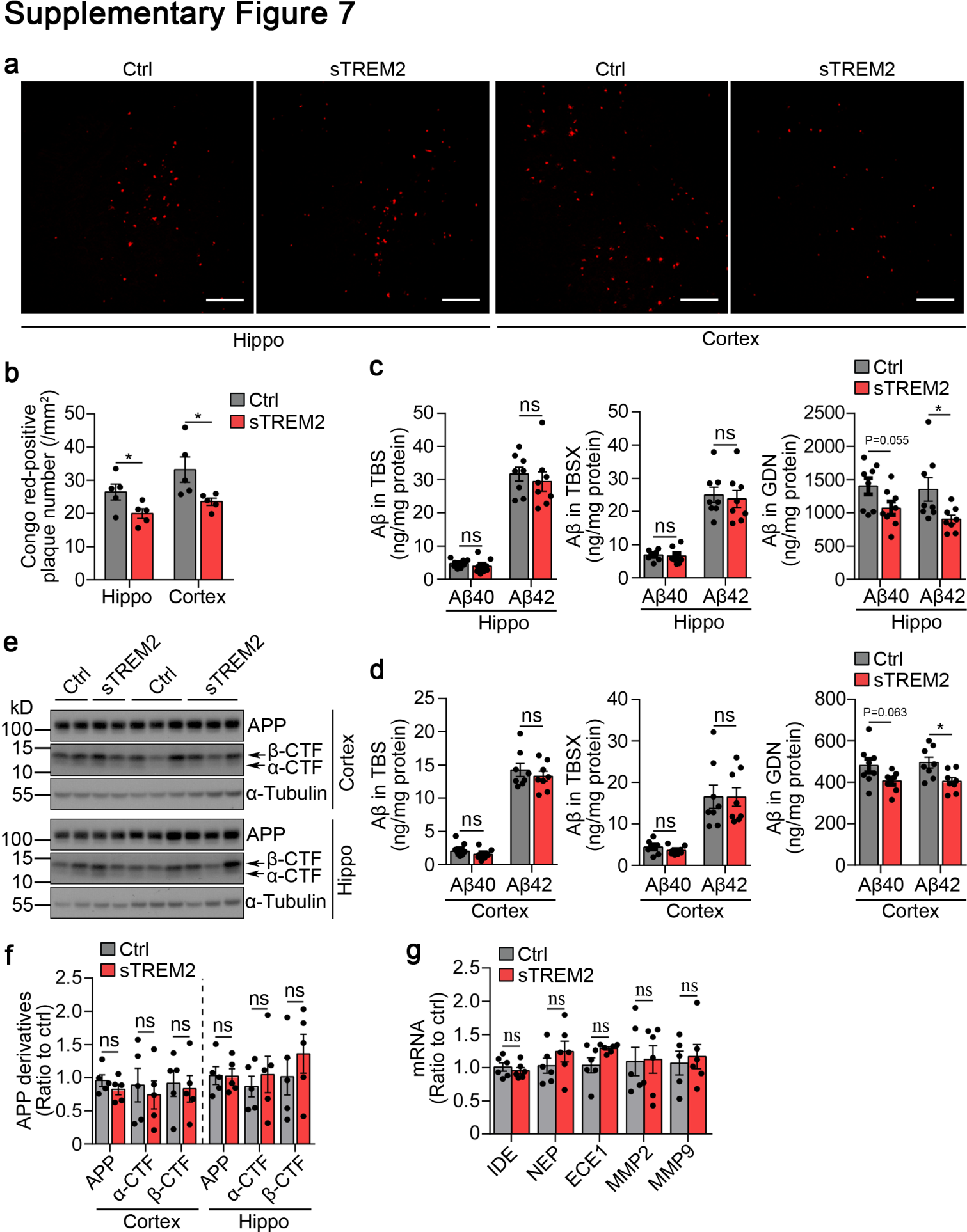
Supplementary Figure 7. AAV-mediated sTREM2 expression decreases the amount of amyloid plaque load but does not affect APP processing nor the levels of Aβ degrading enzymes.**

**a** The 5xFAD mice received either control AAV (Ctrl) or AAV-sTREM2 (sTREM2) were analyzed for amyloid plaque pathology using Congo red staining. Representative images of the hippocampus and cortex of these mice are shown. Original magniﬁcation 10x; scale bar, 200 μm.

**b** Quantitation of the number of Congo red-positive plaques in the hippocampus and cortex of 5xFAD mice injected either with control AAV or AAV-sTREM2 (n = 5 mice per group for hippocampus or cortex, unpaired Student’s t test).

**c**, **d** sTREM2 reduces Aβ levels in the detergent-insoluble (guanidine-HCl, GDN) fractions. Aβ40 and Aβ42 levels in the TBS, TBSX and GDN fractions of hippocampus (c) or cortex (d) region of 5xFAD mice 7 months after receiving control AAV or AAV-sTREM2 were measured by Aβ40 or Aβ42 ELISA (n = 8 mice per group, unpaired Student’s t test).

**e, f** Western blotting analysis of APP and its derivatives, including APP, β-CTF and α-CTF (369 antibody, 1:1000) in the cortex or hippocampus of the AAV-injected 5xFAD mice (n = 5 mice per group, unpaired Student’s t test).

**g** The relative mRNA levels of *IDE*, *NEP*, *ECE1*, *MMP2* and *MMP9* in AAV-injected mice shown as bar graph were determined by quantitative real-time PCR. β-Actin was used as an internal control (n = 5-6 mice per group, unpaired Student’s t test).

All data are presented as mean ± SEM. *, p<0.05; ns, not significant.


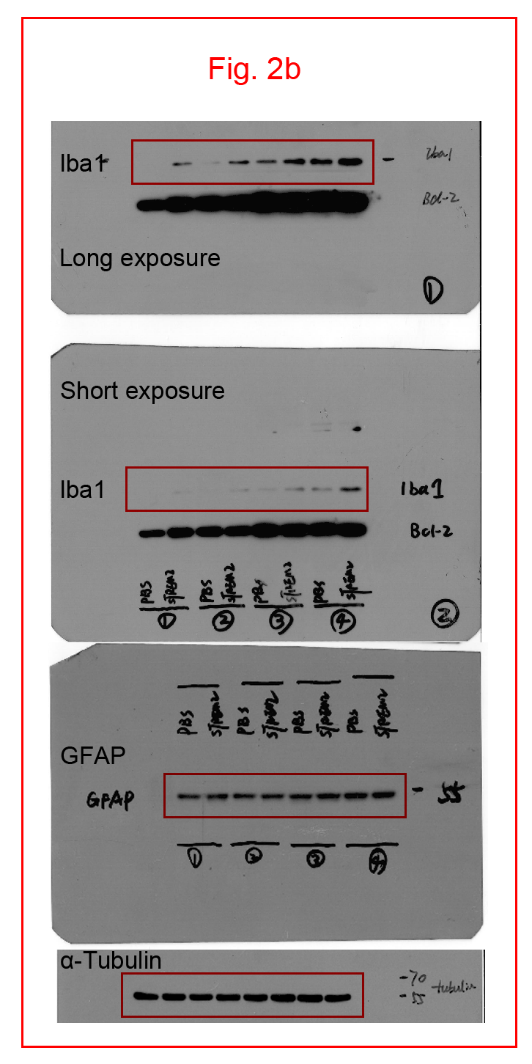

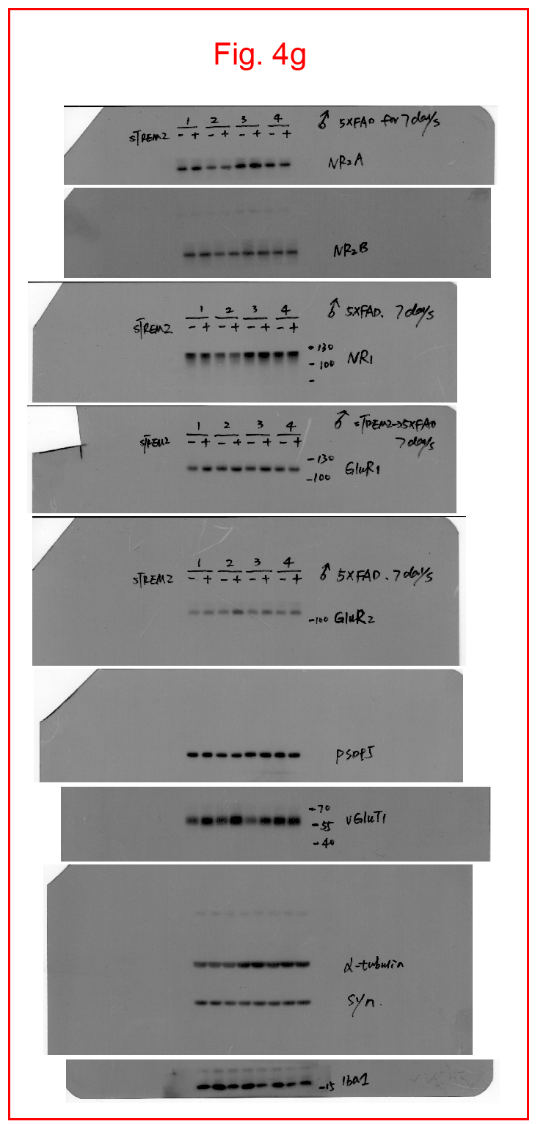

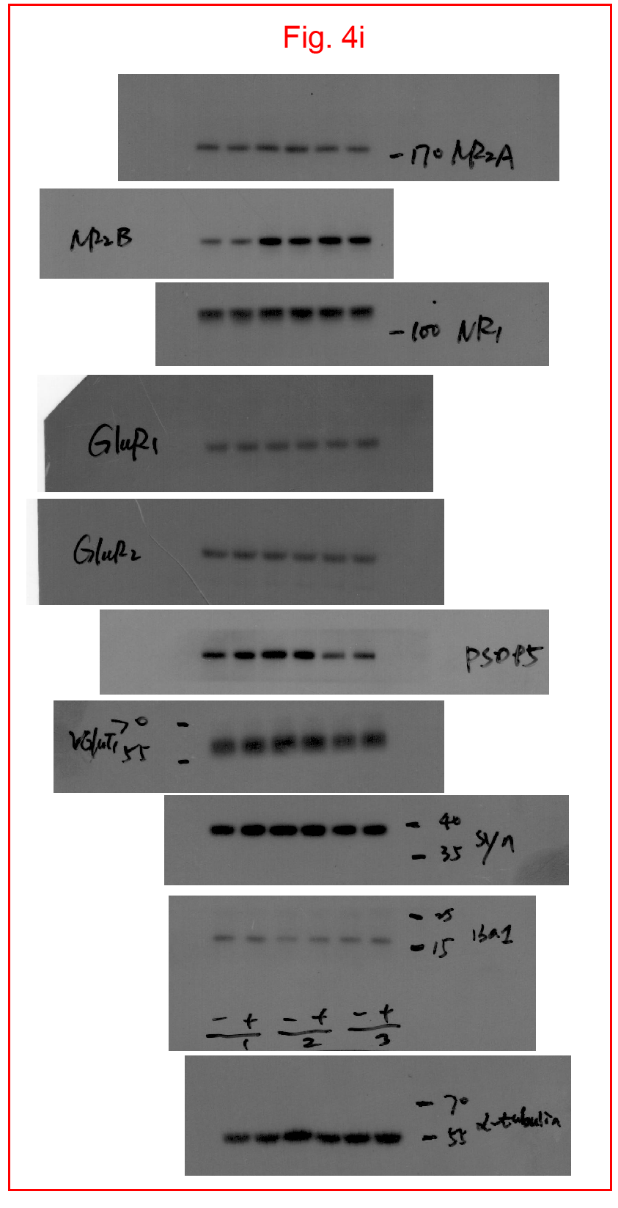

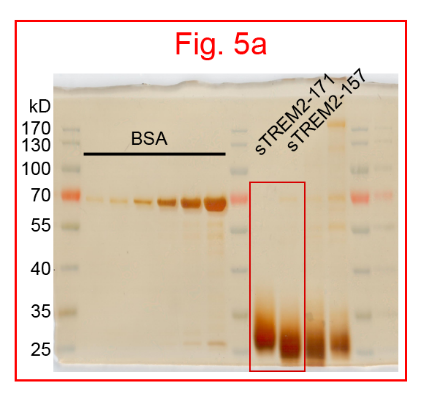

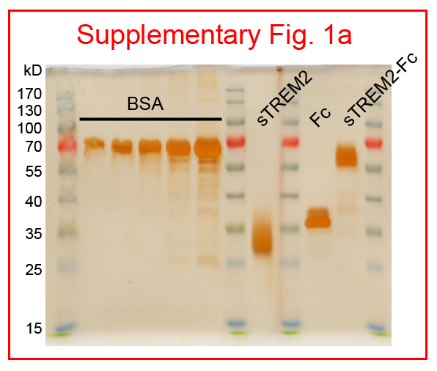

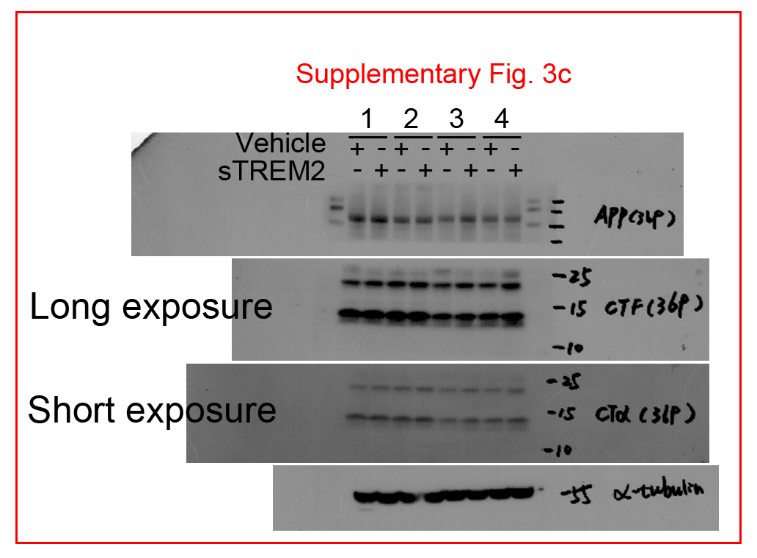

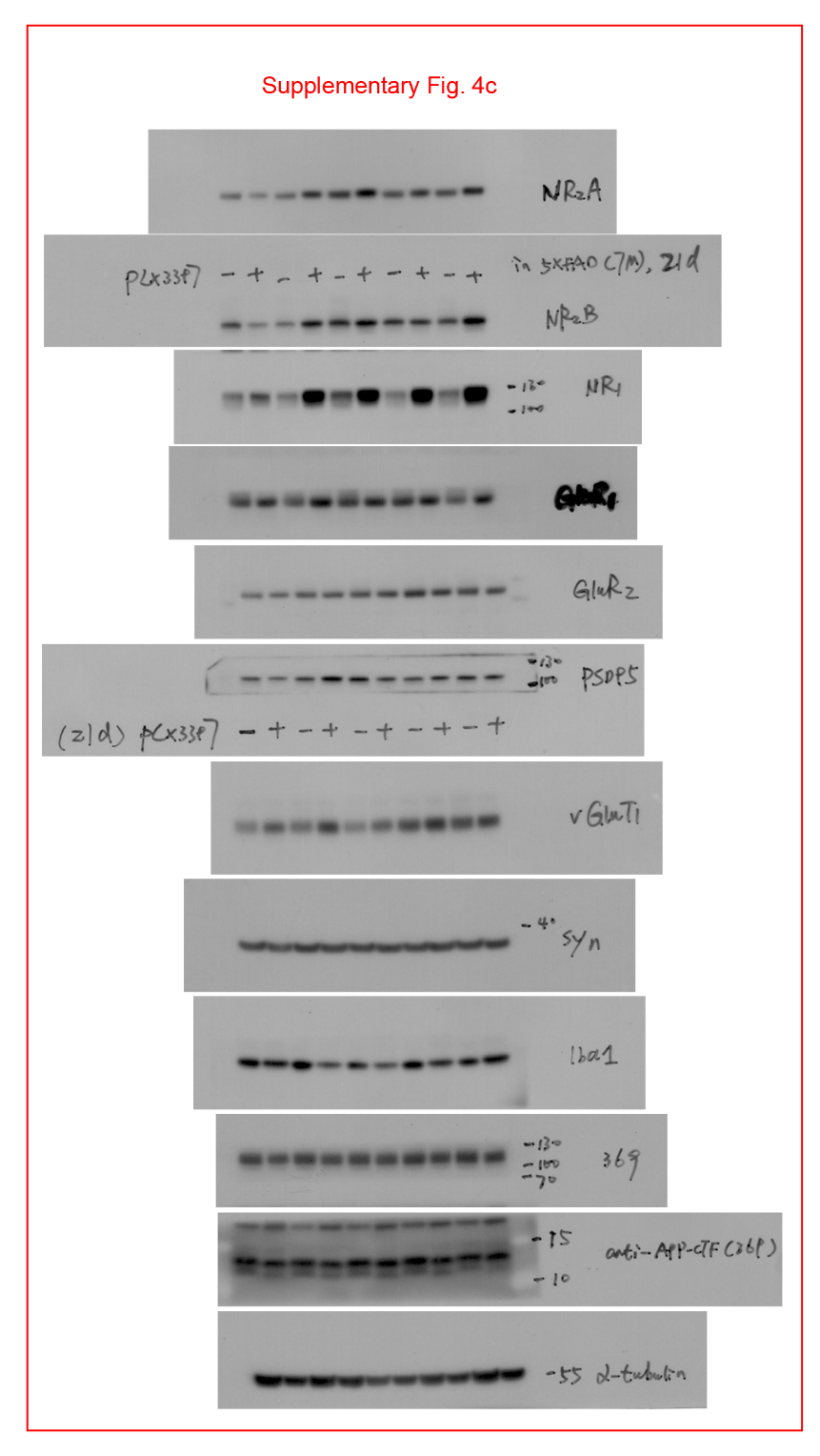

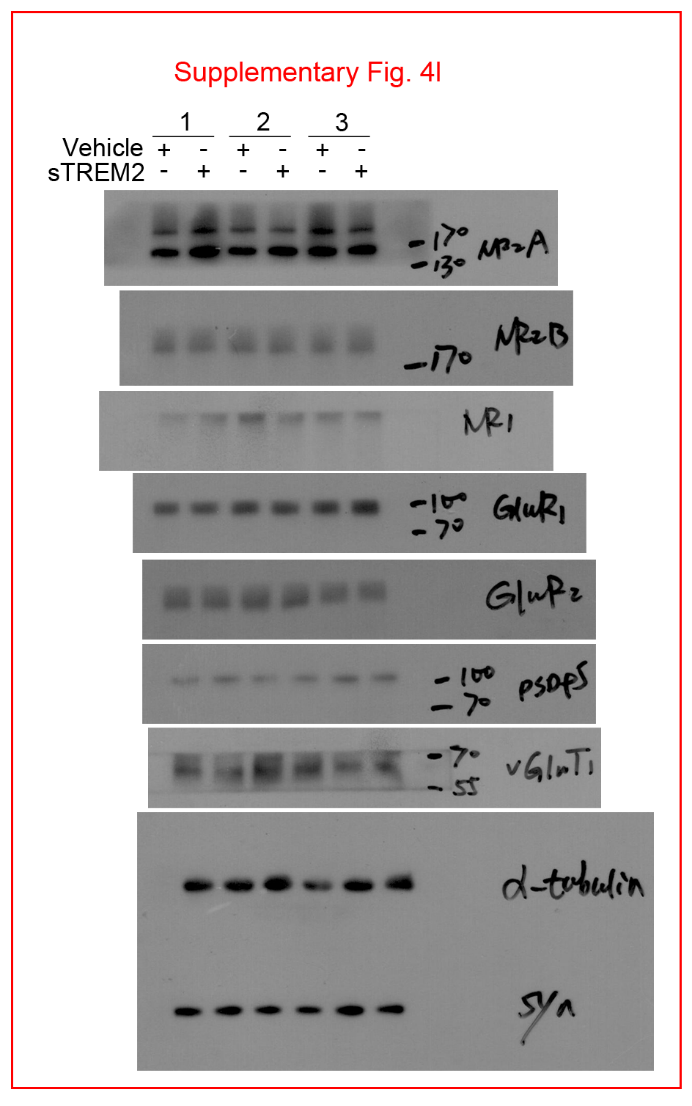

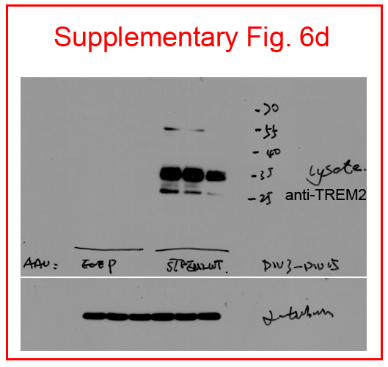

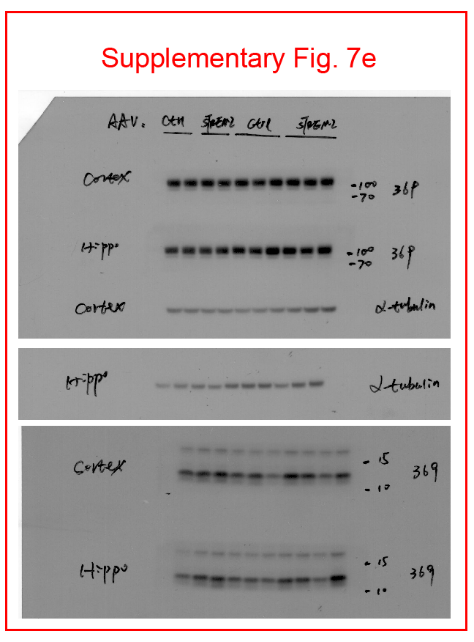

Supplement: Supplementary file 1 — Supplementary Information [file 41467_2019_9118_MOESM1_ESM.docx]
